# Supplementary figures and images for: Erythrocyte invasion-neutralising antibodies prevent Plasmodium falciparum RH5 from binding to basigin-containing membrane protein complexes
Source: eLife. 2023 Oct 5;12:e83681. doi: 10.7554/eLife.83681 (PMC10569788; doi:10.7554/eLife.83681)

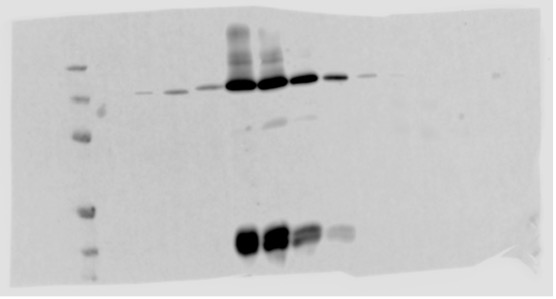

Supplement: Figure 1—source data 2. [file elife-83681-fig1-data2.zip › Figure 1 - source data 2/1a/lower middle.jpg]

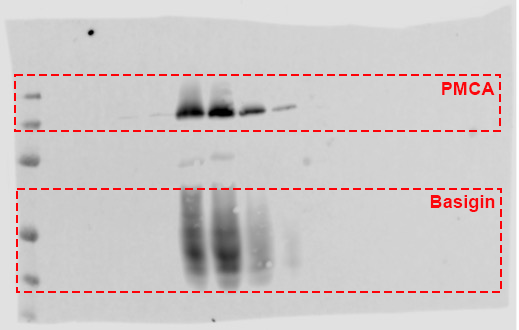

Supplement: Figure 1—source data 2. [file elife-83681-fig1-data2.zip › Figure 1 - source data 2/1a/upper and upper middle markup.tif]

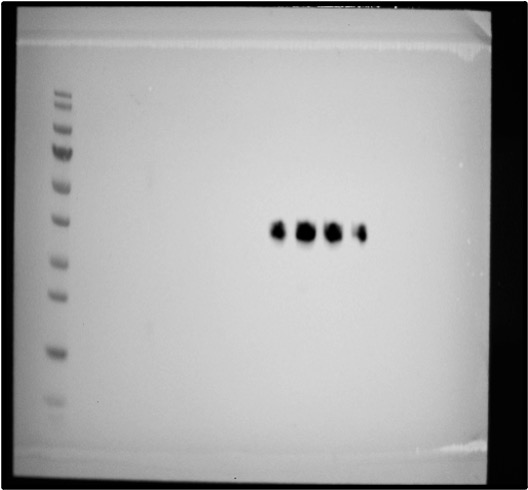

Supplement: Figure 1—source data 2. [file elife-83681-fig1-data2.zip › Figure 1 - source data 2/1a/lower.jpg]

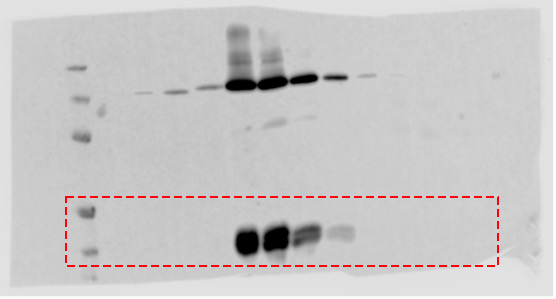

Supplement: Figure 1—source data 2. [file elife-83681-fig1-data2.zip › Figure 1 - source data 2/1a/lower middle markup.tif]

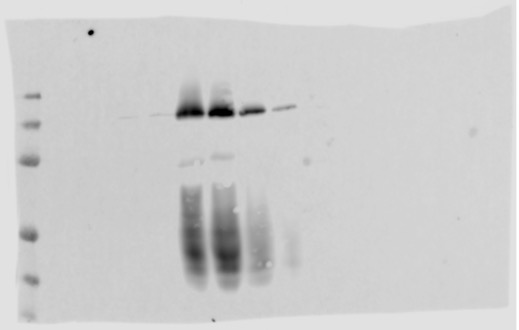

Supplement: Figure 1—source data 2. [file elife-83681-fig1-data2.zip › Figure 1 - source data 2/1a/upper and upper middle.jpg]

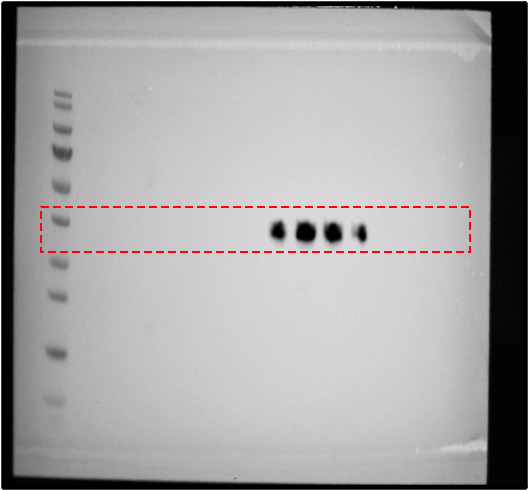

Supplement: Figure 1—source data 2. [file elife-83681-fig1-data2.zip › Figure 1 - source data 2/1a/lower markup.tif]

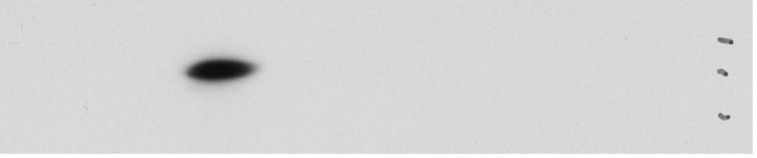

Supplement: Figure 1—source data 2. [file elife-83681-fig1-data2.zip › Figure 1 - source data 2/1b/upper panel PMCA.jpg]

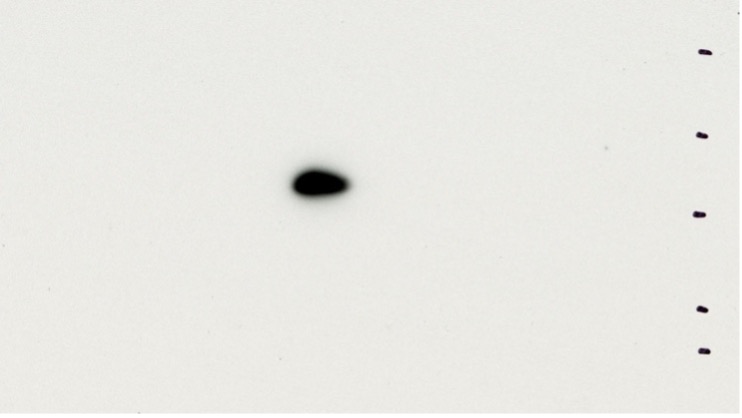

Supplement: Figure 1—source data 2. [file elife-83681-fig1-data2.zip › Figure 1 - source data 2/1b/lower panel MCT1.jpg]

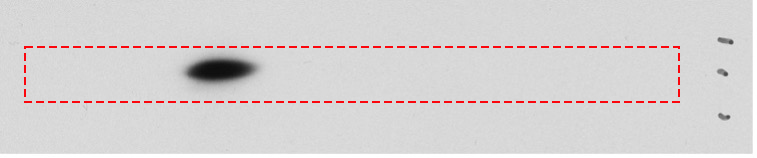

Supplement: Figure 1—source data 2. [file elife-83681-fig1-data2.zip › Figure 1 - source data 2/1b/upper panel PMCA markup.jpg]

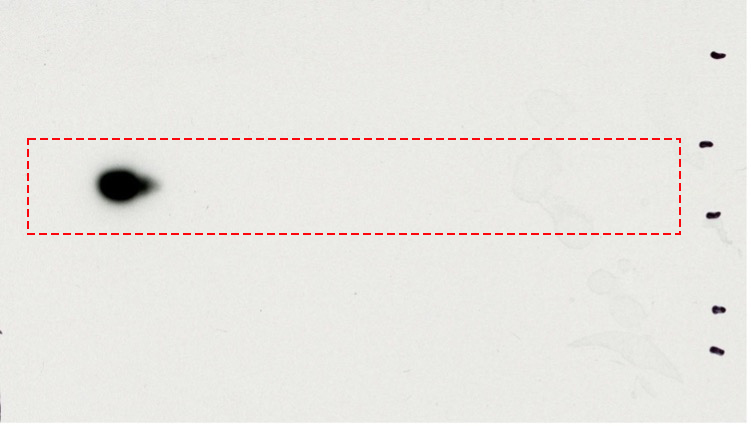

Supplement: Figure 1—source data 2. [file elife-83681-fig1-data2.zip › Figure 1 - source data 2/1b/upper panel MCT1 markup.jpg]

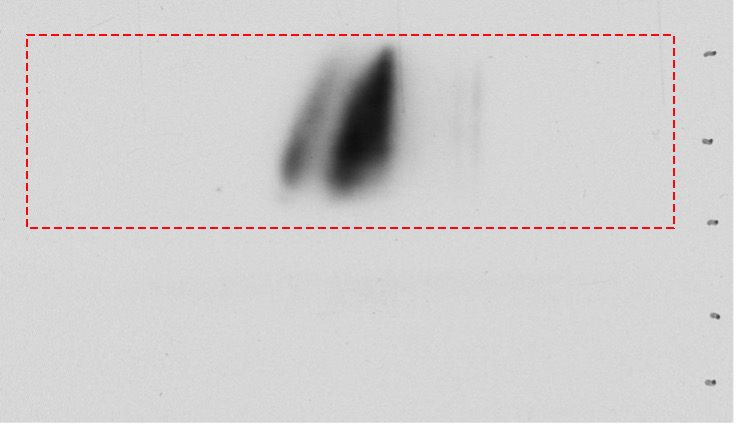

Supplement: Figure 1—source data 2. [file elife-83681-fig1-data2.zip › Figure 1 - source data 2/1b/lower panel basigin markup.jpg]

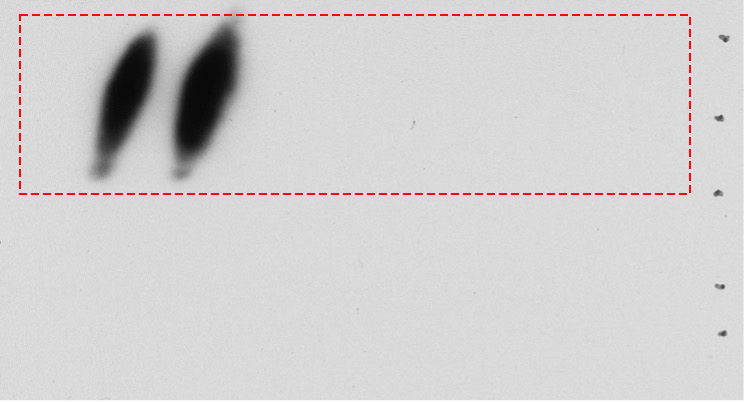

Supplement: Figure 1—source data 2. [file elife-83681-fig1-data2.zip › Figure 1 - source data 2/1b/upper panel basigin markup.jpg]

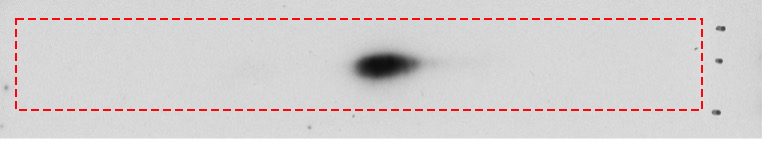

Supplement: Figure 1—source data 2. [file elife-83681-fig1-data2.zip › Figure 1 - source data 2/1b/lower panel PMCA markup.jpg]

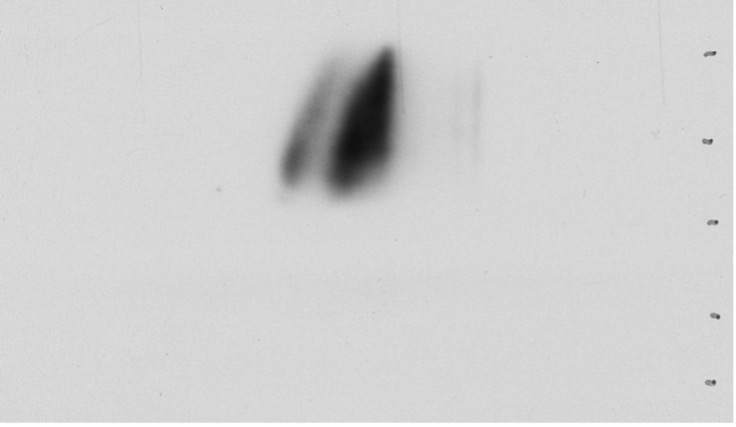

Supplement: Figure 1—source data 2. [file elife-83681-fig1-data2.zip › Figure 1 - source data 2/1b/lower panel basigin.jpg]

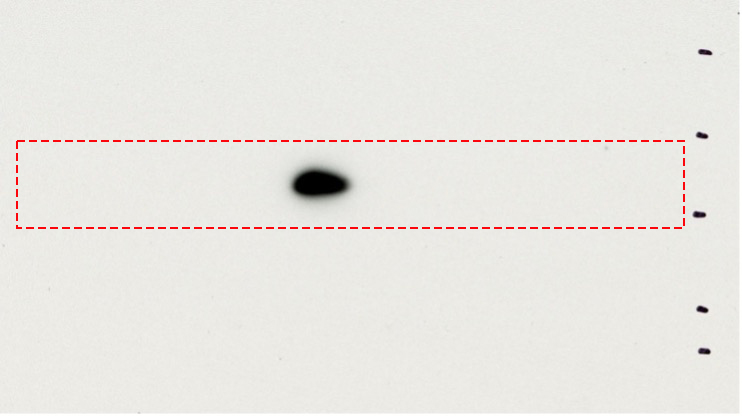

Supplement: Figure 1—source data 2. [file elife-83681-fig1-data2.zip › Figure 1 - source data 2/1b/lower panel MCT1 markup.jpg]

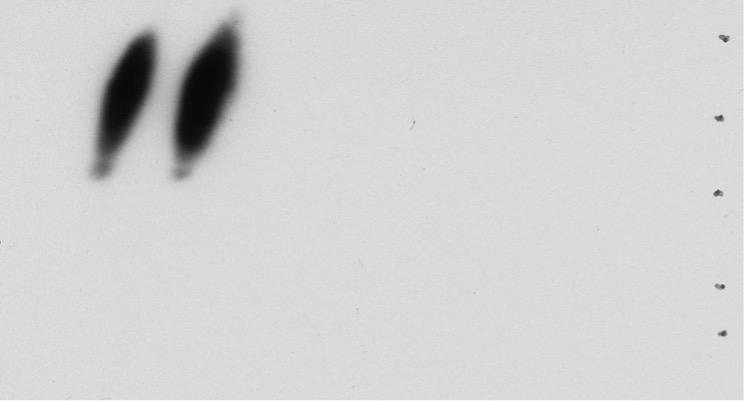

Supplement: Figure 1—source data 2. [file elife-83681-fig1-data2.zip › Figure 1 - source data 2/1b/upper panel basigin.jpg]

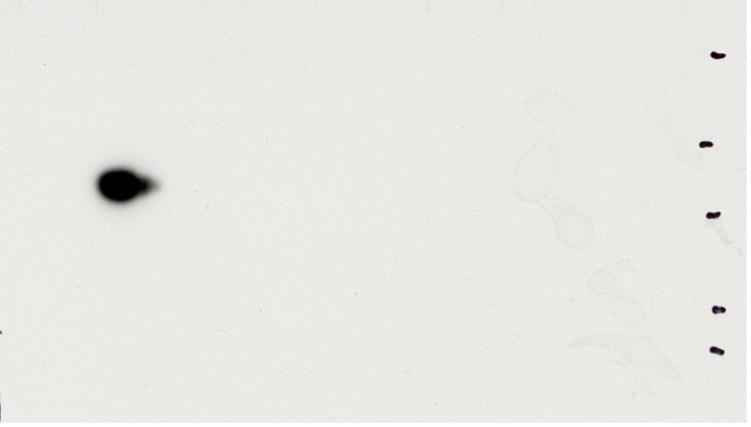

Supplement: Figure 1—source data 2. [file elife-83681-fig1-data2.zip › Figure 1 - source data 2/1b/upper panel MCT1.jpg]

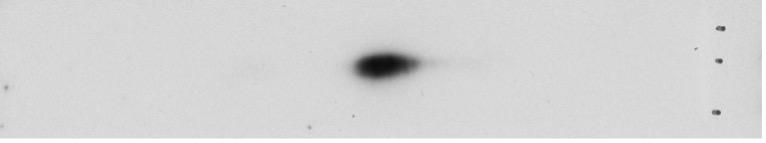

Supplement: Figure 1—source data 2. [file elife-83681-fig1-data2.zip › Figure 1 - source data 2/1b/lower panel PMCA.jpg]

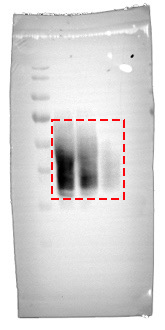

Supplement: Figure 1—source data 2. [file elife-83681-fig1-data2.zip › Figure 1 - source data 2/1c/lower middle markup.jpg]

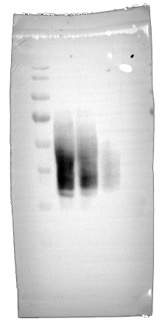

Supplement: Figure 1—source data 2. [file elife-83681-fig1-data2.zip › Figure 1 - source data 2/1c/lower middle.jpg]

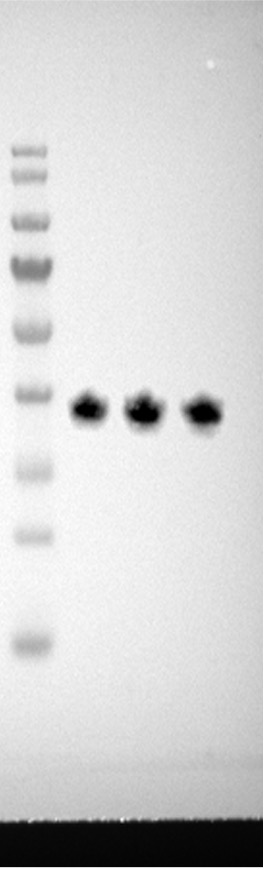

Supplement: Figure 1—source data 2. [file elife-83681-fig1-data2.zip › Figure 1 - source data 2/1c/lower panel.jpg]

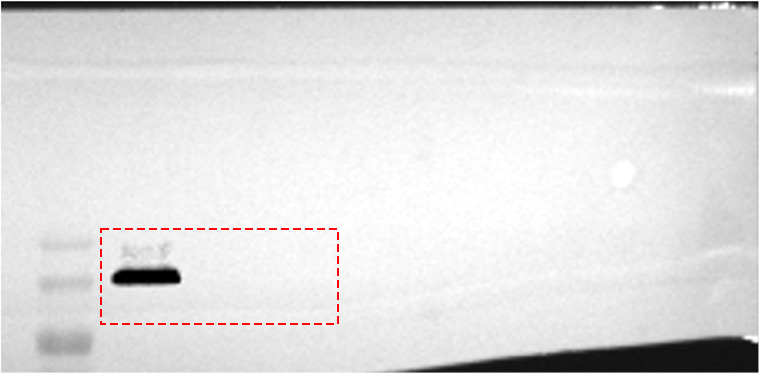

Supplement: Figure 1—source data 2. [file elife-83681-fig1-data2.zip › Figure 1 - source data 2/1c/upper panel markup.jpg]

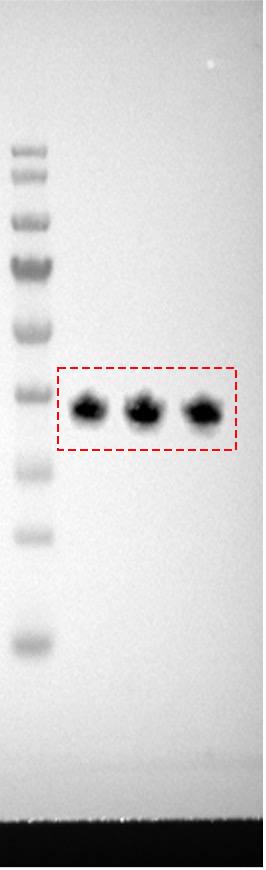

Supplement: Figure 1—source data 2. [file elife-83681-fig1-data2.zip › Figure 1 - source data 2/1c/lower panel markup.tif]

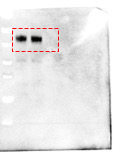

Supplement: Figure 1—source data 2. [file elife-83681-fig1-data2.zip › Figure 1 - source data 2/1c/upper middle markup.tif]

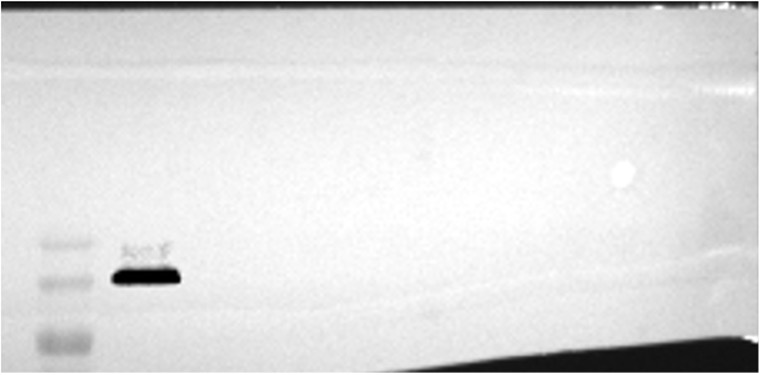

Supplement: Figure 1—source data 2. [file elife-83681-fig1-data2.zip › Figure 1 - source data 2/1c/upper panel.jpg]

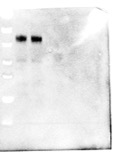

Supplement: Figure 1—source data 2. [file elife-83681-fig1-data2.zip › Figure 1 - source data 2/1c/upper middle.jpg]

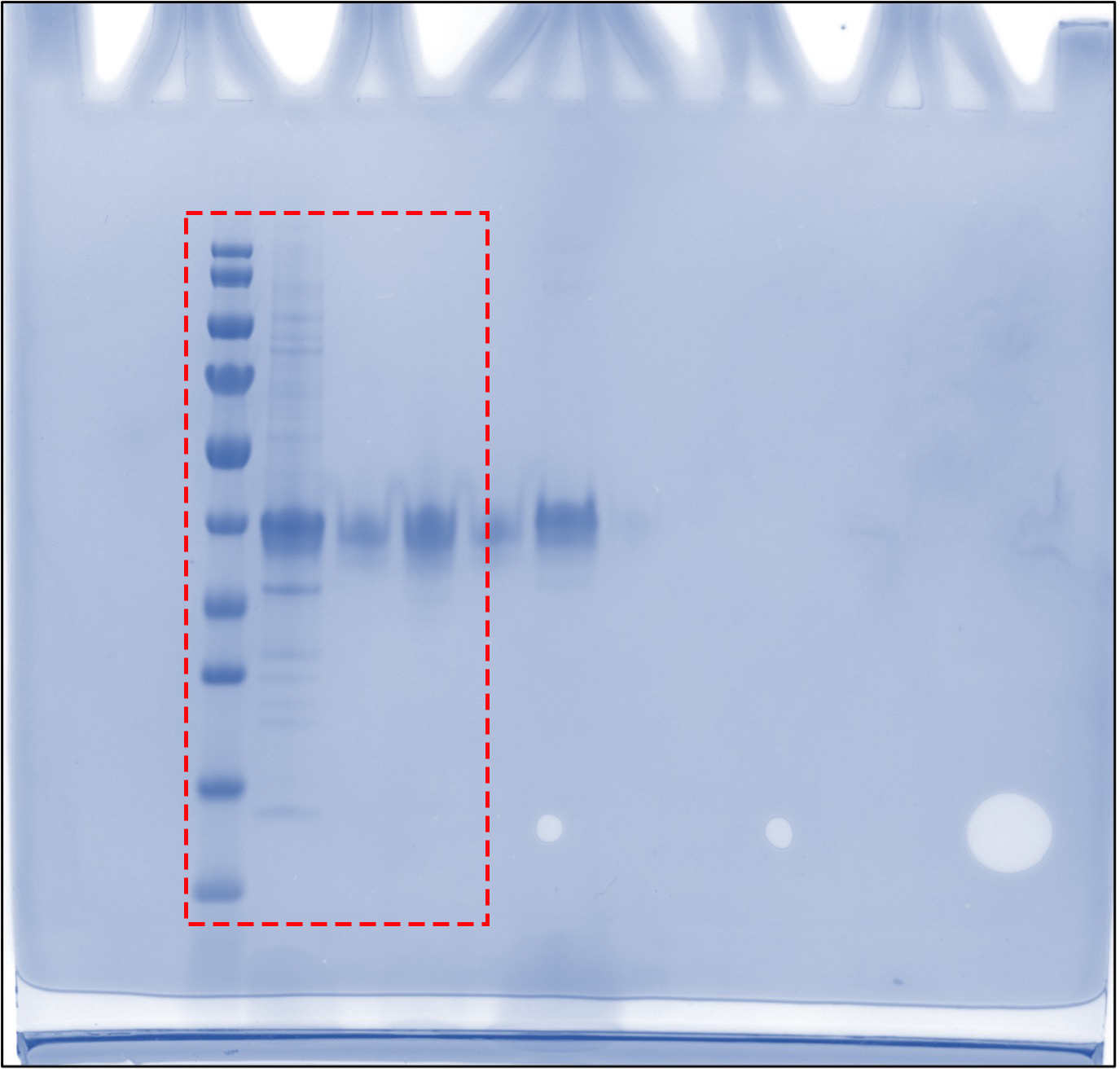

Supplement: Figure 1—figure supplement 1—source data 2. [file elife-83681-fig1-figsupp1-data2.zip › Figure 1 - figure supplement 1 - source data 2/a/a markup.jpg]

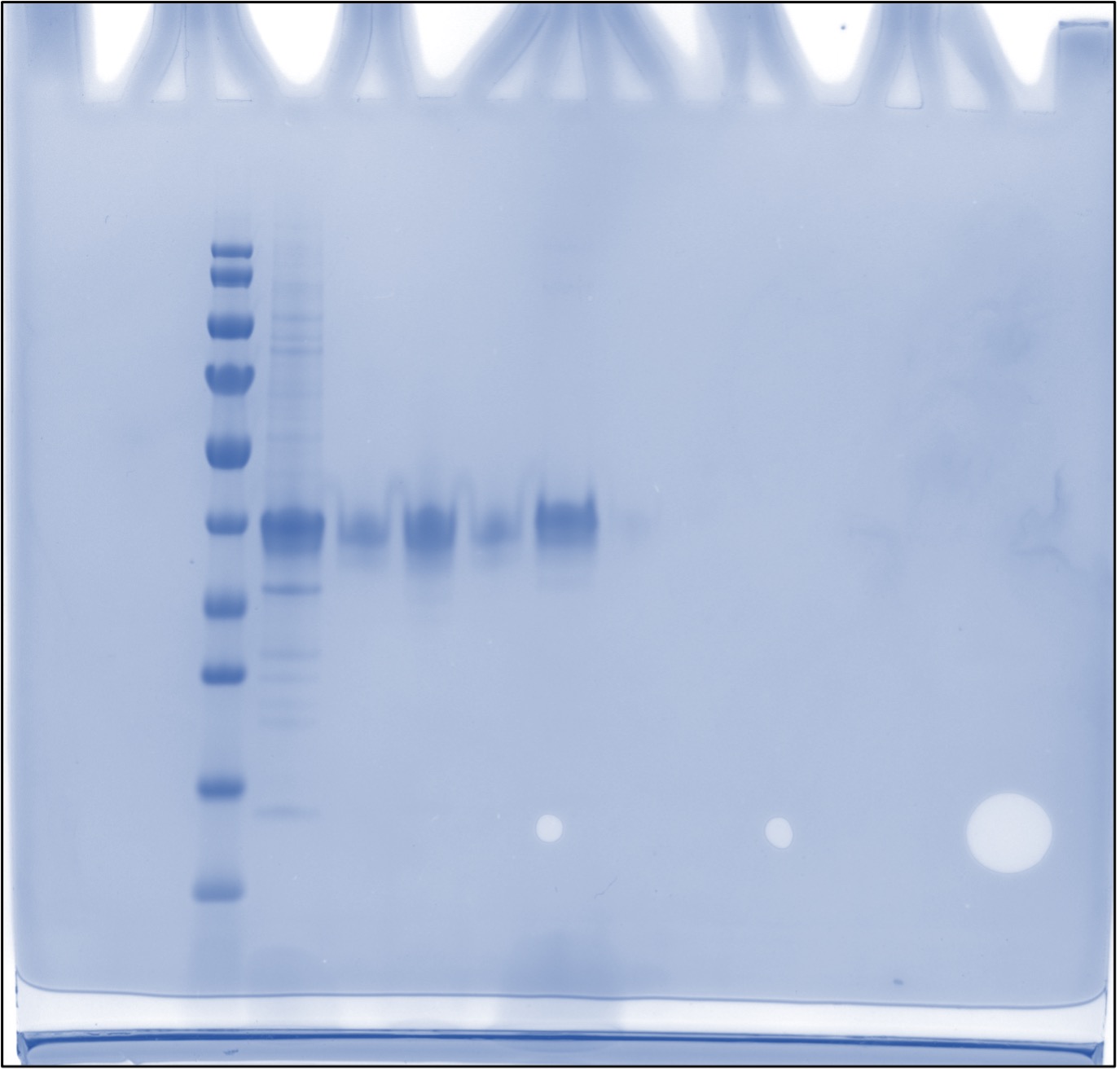

Supplement: Figure 1—figure supplement 1—source data 2. [file elife-83681-fig1-figsupp1-data2.zip › Figure 1 - figure supplement 1 - source data 2/a/a.jpg]

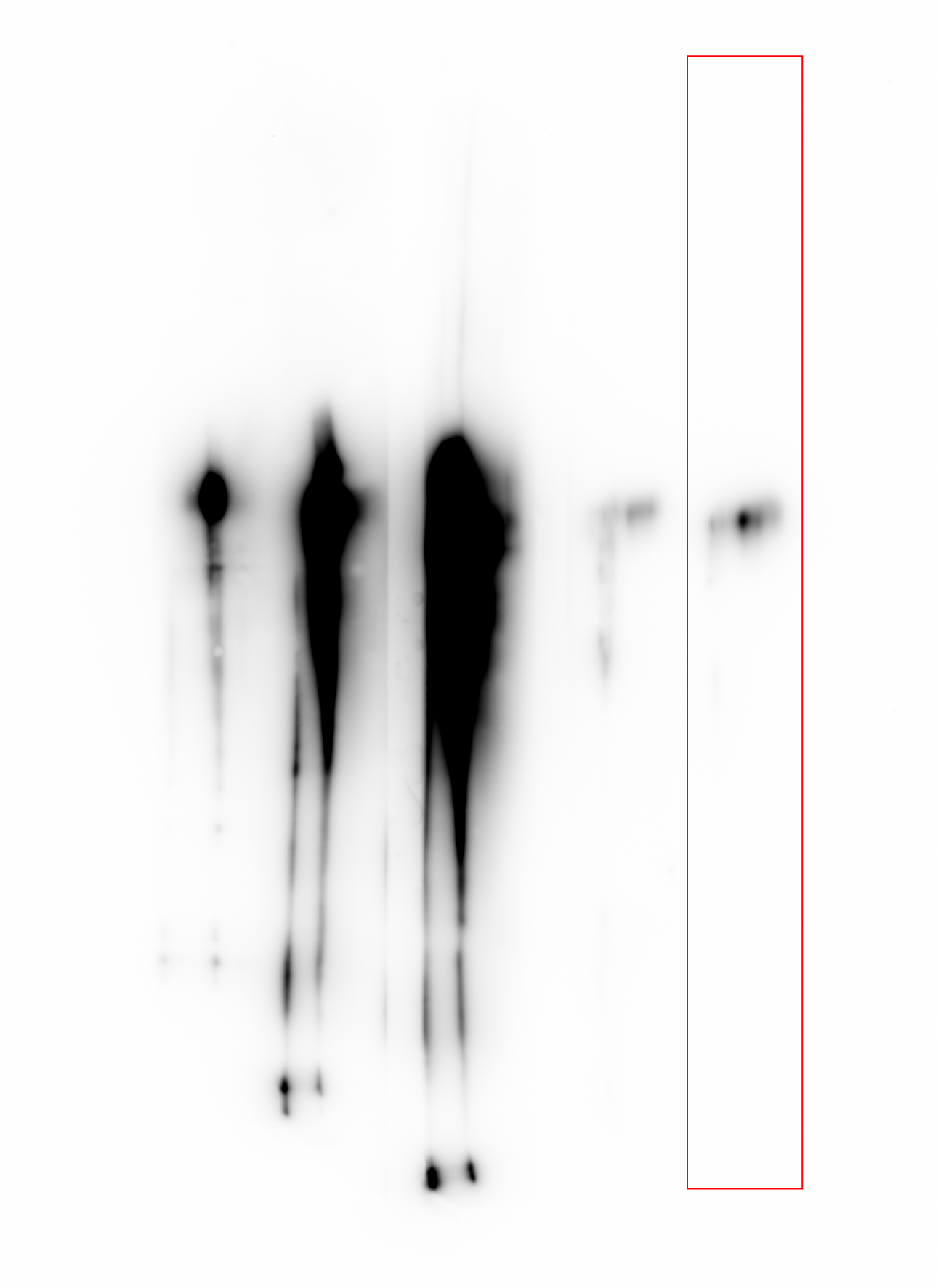

Supplement: Figure 1—figure supplement 1—source data 2. [file elife-83681-fig1-figsupp1-data2.zip › Figure 1 - figure supplement 1 - source data 2/c/PMCA markup.tif]

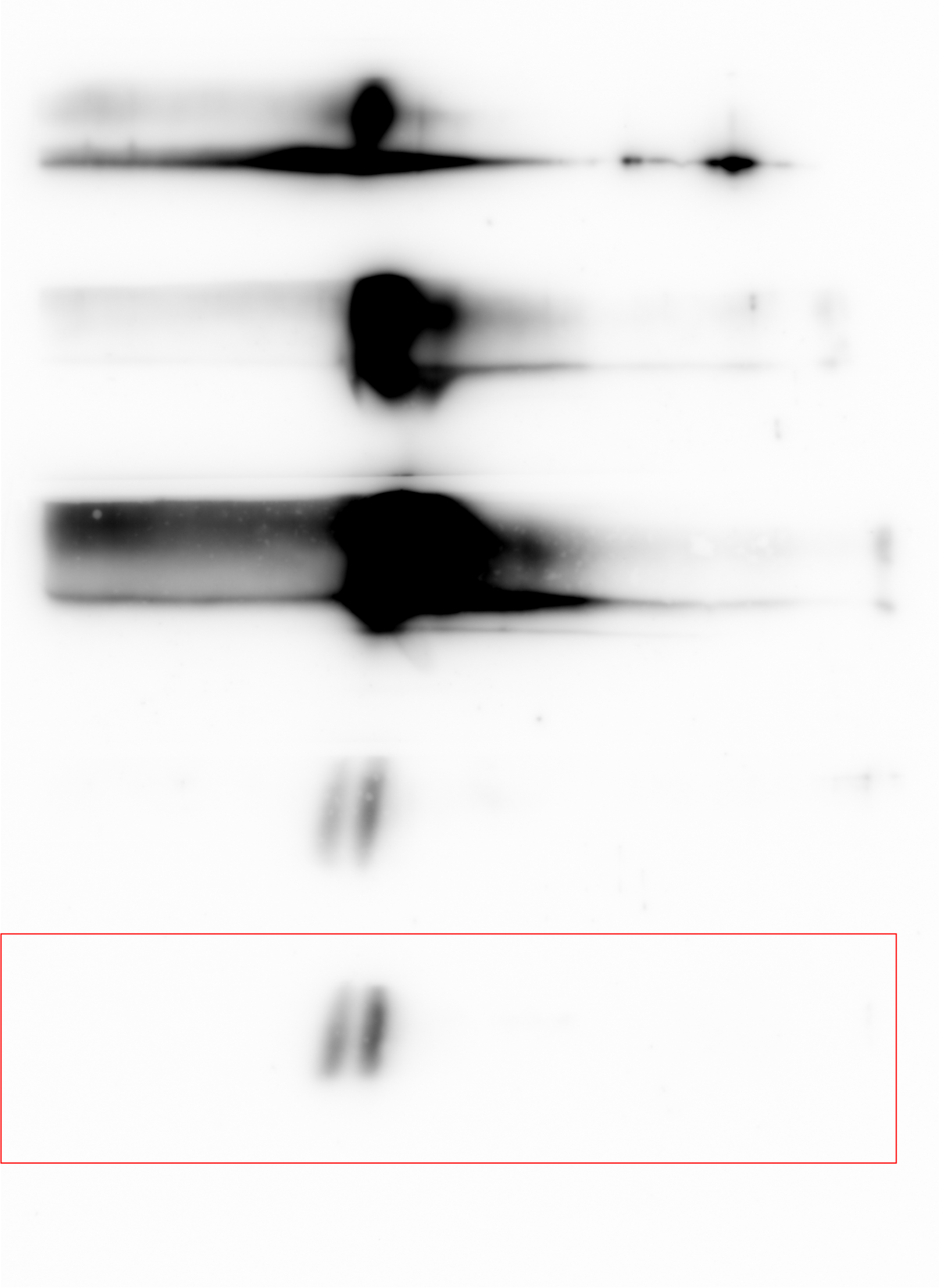

Supplement: Figure 1—figure supplement 1—source data 2. [file elife-83681-fig1-figsupp1-data2.zip › Figure 1 - figure supplement 1 - source data 2/c/basigin markup.tif]

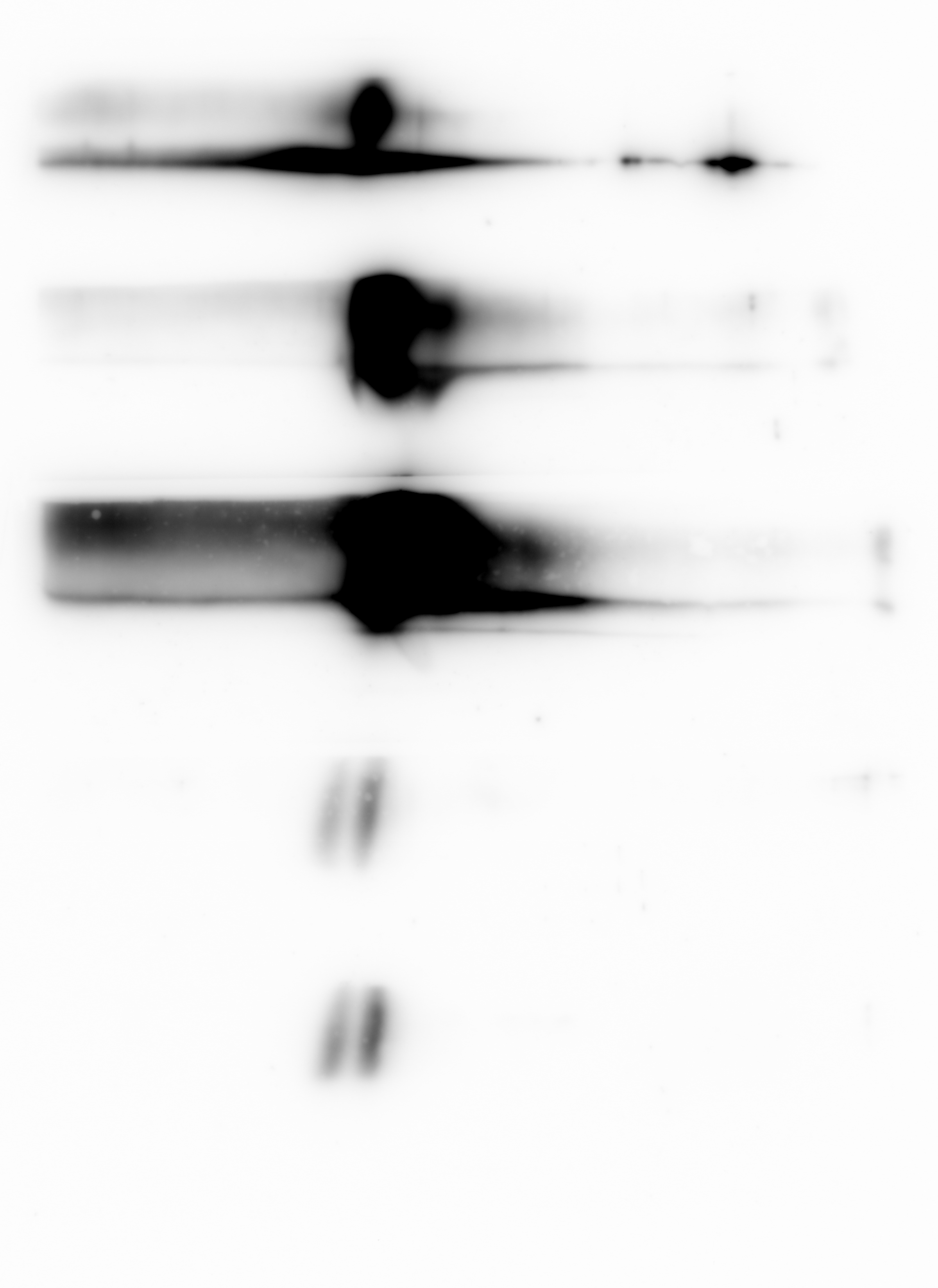

Supplement: Figure 1—figure supplement 1—source data 2. [file elife-83681-fig1-figsupp1-data2.zip › Figure 1 - figure supplement 1 - source data 2/c/basigin.tif]

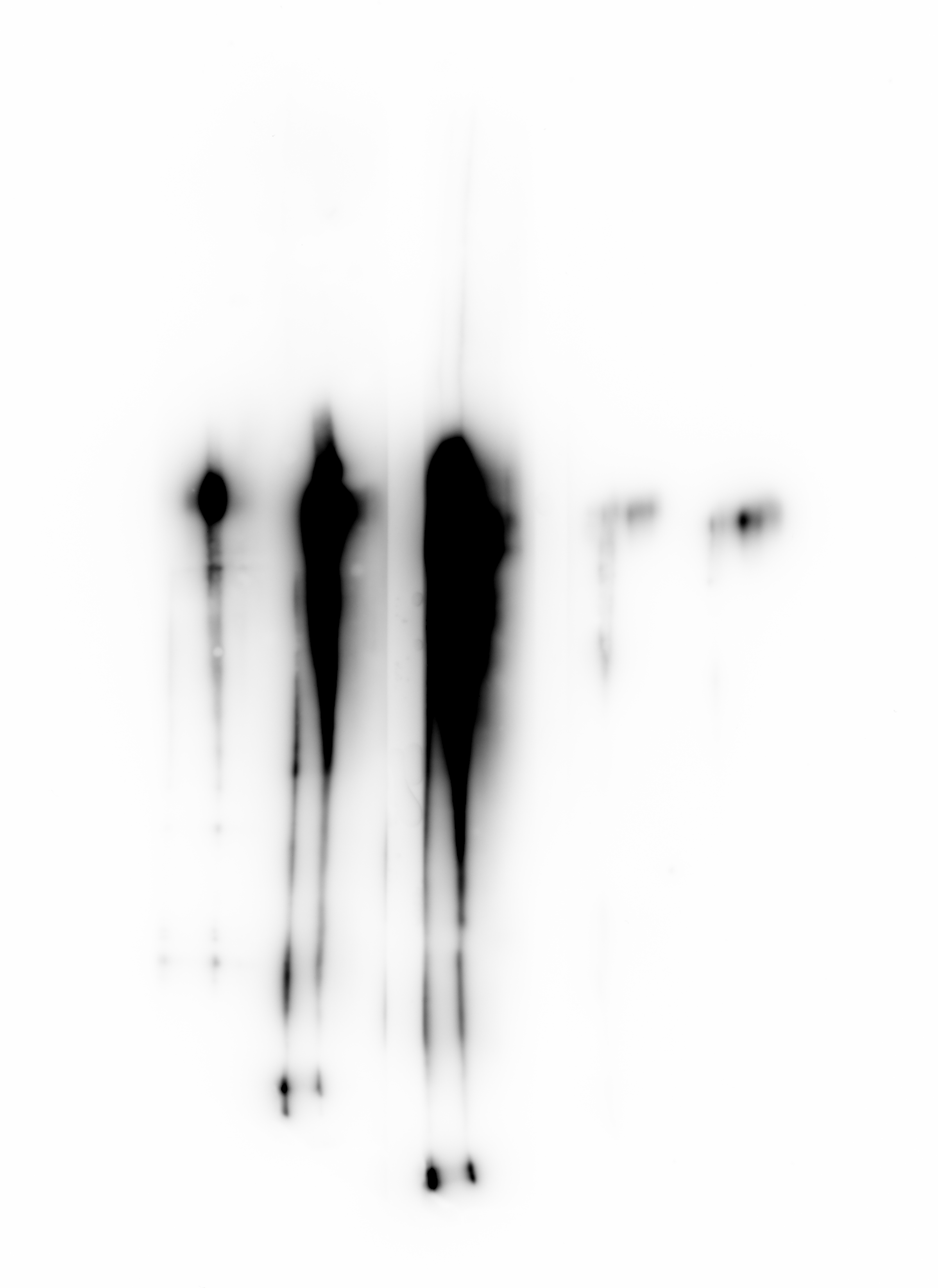

Supplement: Figure 1—figure supplement 1—source data 2. [file elife-83681-fig1-figsupp1-data2.zip › Figure 1 - figure supplement 1 - source data 2/c/PMCA.tif]

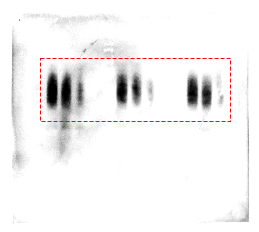

Supplement: Figure 1—figure supplement 1—source data 2. [file elife-83681-fig1-figsupp1-data2.zip › Figure 1 - figure supplement 1 - source data 2/d/lower middle markup.png]

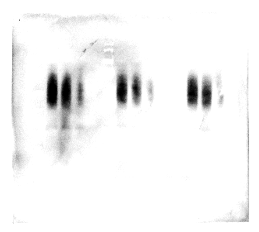

Supplement: Figure 1—figure supplement 1—source data 2. [file elife-83681-fig1-figsupp1-data2.zip › Figure 1 - figure supplement 1 - source data 2/d/lower middle.jpg]

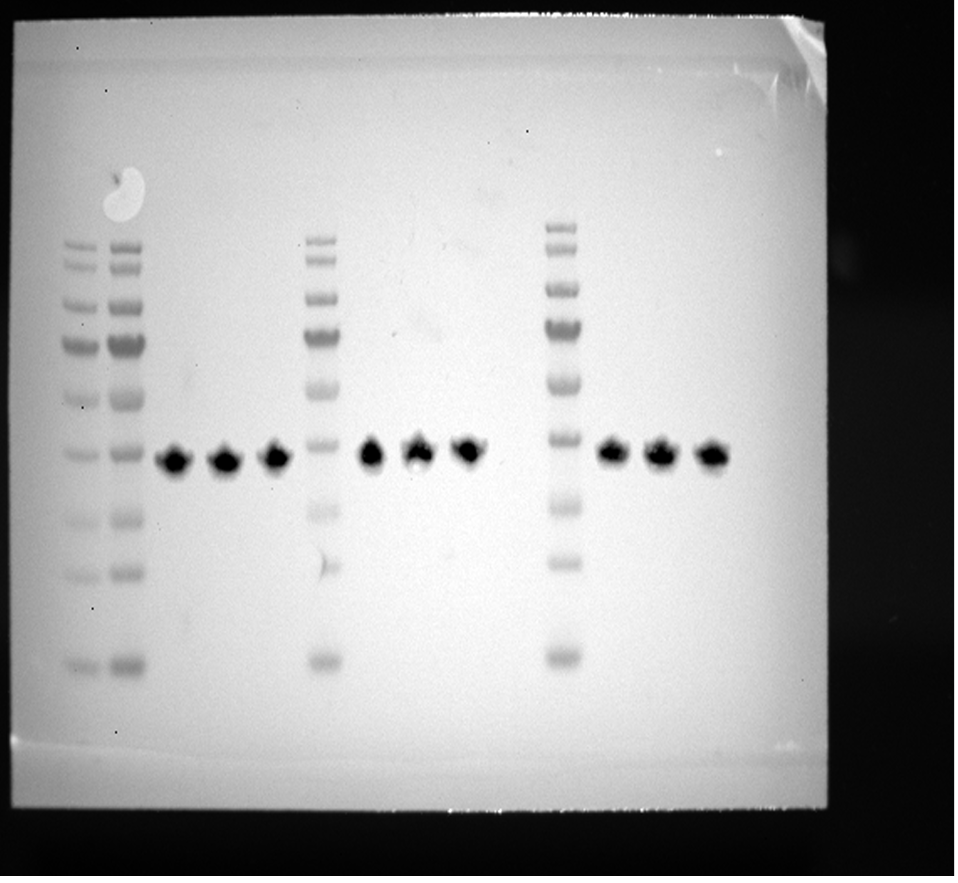

Supplement: Figure 1—figure supplement 1—source data 2. [file elife-83681-fig1-figsupp1-data2.zip › Figure 1 - figure supplement 1 - source data 2/d/lower panel.jpg]

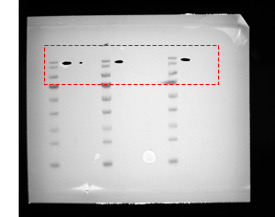

Supplement: Figure 1—figure supplement 1—source data 2. [file elife-83681-fig1-figsupp1-data2.zip › Figure 1 - figure supplement 1 - source data 2/d/upper panel markup.jpg]

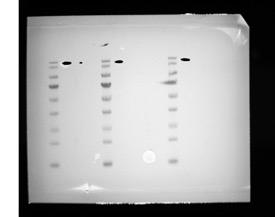

Supplement: Figure 1—figure supplement 1—source data 2. [file elife-83681-fig1-figsupp1-data2.zip › Figure 1 - figure supplement 1 - source data 2/d/upper panel.jpg]

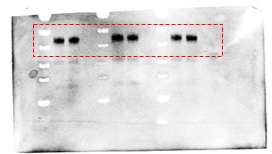

Supplement: Figure 1—figure supplement 1—source data 2. [file elife-83681-fig1-figsupp1-data2.zip › Figure 1 - figure supplement 1 - source data 2/d/upper middle markup.jpg]

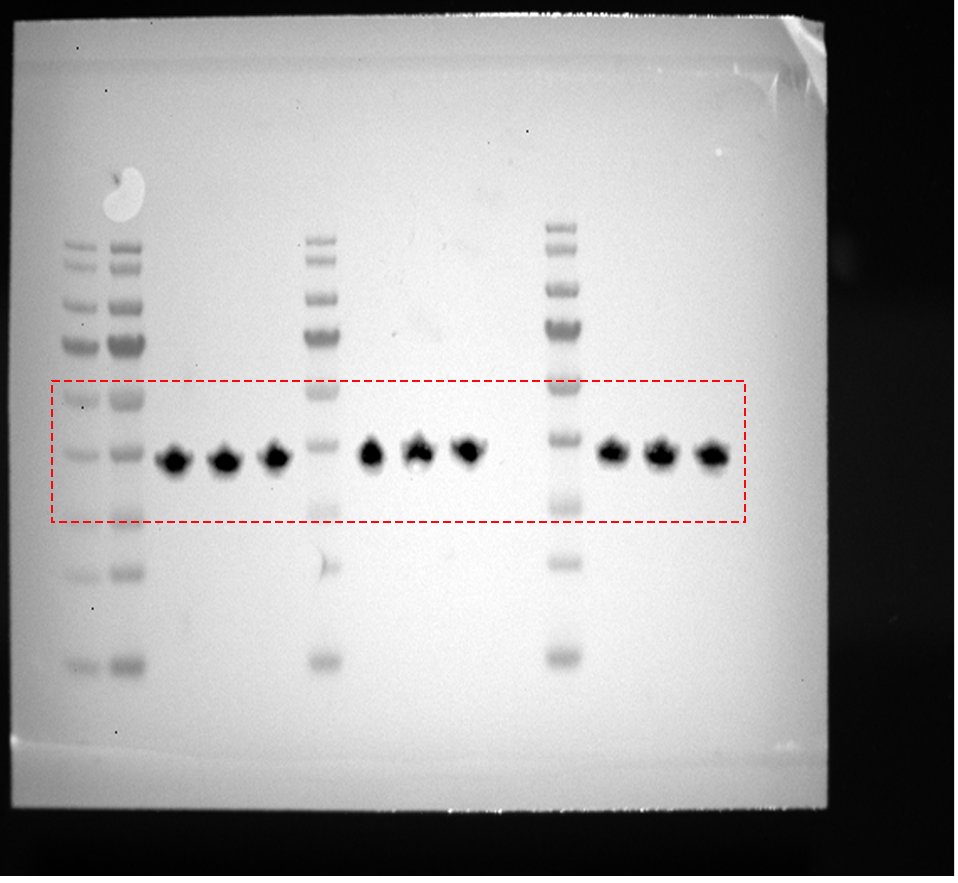

Supplement: Figure 1—figure supplement 1—source data 2. [file elife-83681-fig1-figsupp1-data2.zip › Figure 1 - figure supplement 1 - source data 2/d/lower panel markup.png]

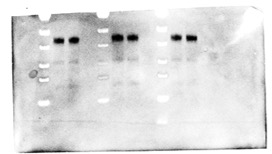

Supplement: Figure 1—figure supplement 1—source data 2. [file elife-83681-fig1-figsupp1-data2.zip › Figure 1 - figure supplement 1 - source data 2/d/upper middle.jpg]

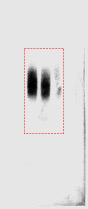

Supplement: Figure 1—figure supplement 1—source data 2. [file elife-83681-fig1-figsupp1-data2.zip › Figure 1 - figure supplement 1 - source data 2/e/1e markup.jpg]

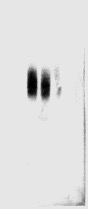

Supplement: Figure 1—figure supplement 1—source data 2. [file elife-83681-fig1-figsupp1-data2.zip › Figure 1 - figure supplement 1 - source data 2/e/1e.jpg]

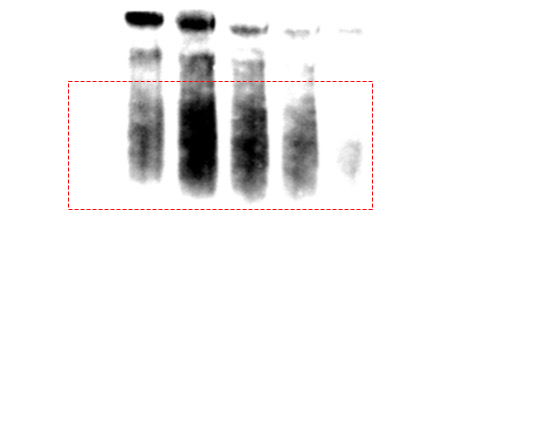

Supplement: Figure 1—figure supplement 2—source data 2. [file elife-83681-fig1-figsupp2-data2.zip › Figure 1 - figure supplement 2 - source data 2/lower middle markup.jpg]

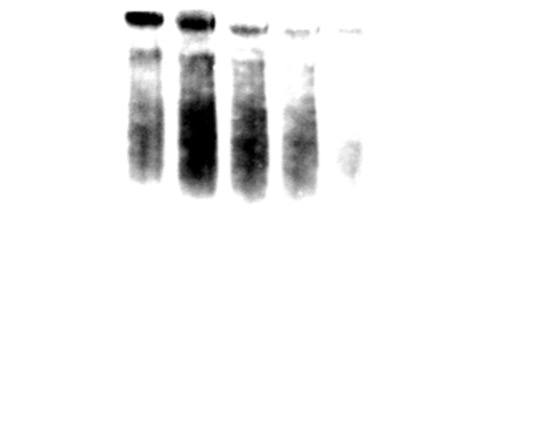

Supplement: Figure 1—figure supplement 2—source data 2. [file elife-83681-fig1-figsupp2-data2.zip › Figure 1 - figure supplement 2 - source data 2/lower middle.jpg]

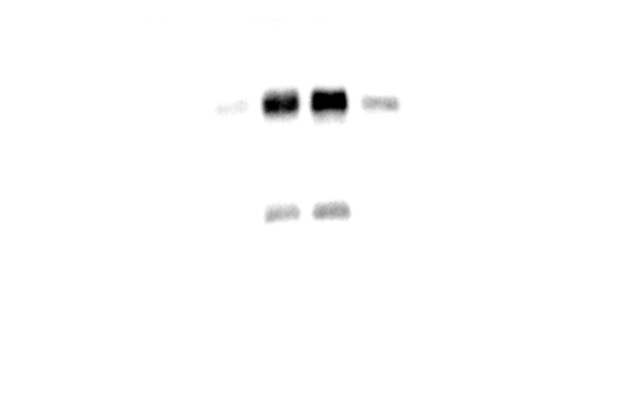

Supplement: Figure 1—figure supplement 2—source data 2. [file elife-83681-fig1-figsupp2-data2.zip › Figure 1 - figure supplement 2 - source data 2/upper left.jpg]

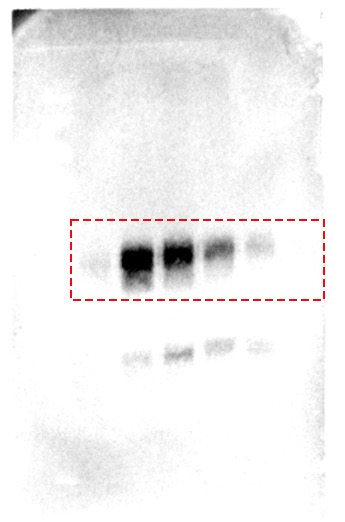

Supplement: Figure 1—figure supplement 2—source data 2. [file elife-83681-fig1-figsupp2-data2.zip › Figure 1 - figure supplement 2 - source data 2/lower left markup.jpg]

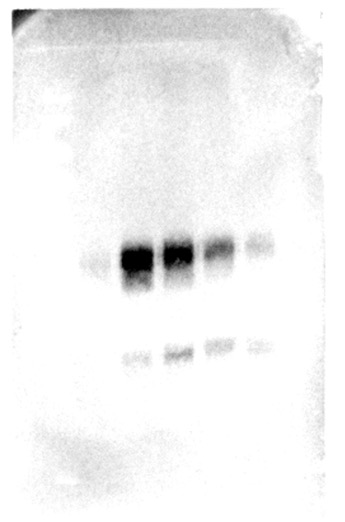

Supplement: Figure 1—figure supplement 2—source data 2. [file elife-83681-fig1-figsupp2-data2.zip › Figure 1 - figure supplement 2 - source data 2/lower left.jpg]

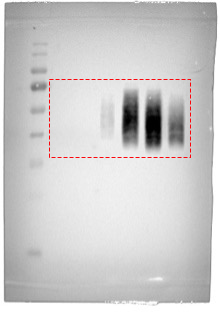

Supplement: Figure 1—figure supplement 2—source data 2. [file elife-83681-fig1-figsupp2-data2.zip › Figure 1 - figure supplement 2 - source data 2/upper middle markup.jpg]

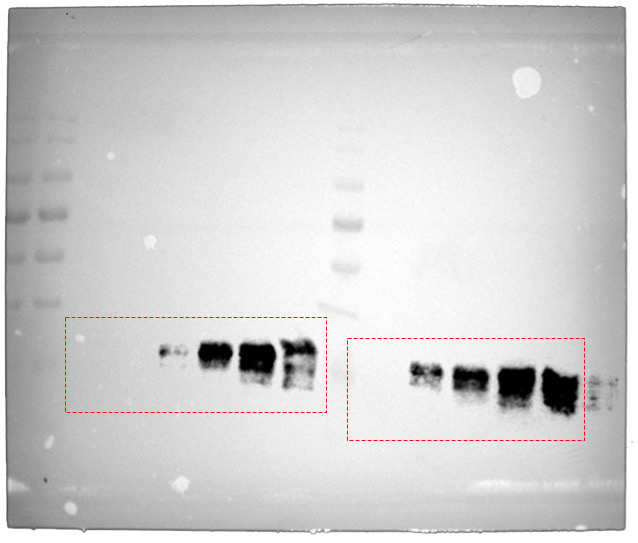

Supplement: Figure 1—figure supplement 2—source data 2. [file elife-83681-fig1-figsupp2-data2.zip › Figure 1 - figure supplement 2 - source data 2/upper and lower right markup.jpg]

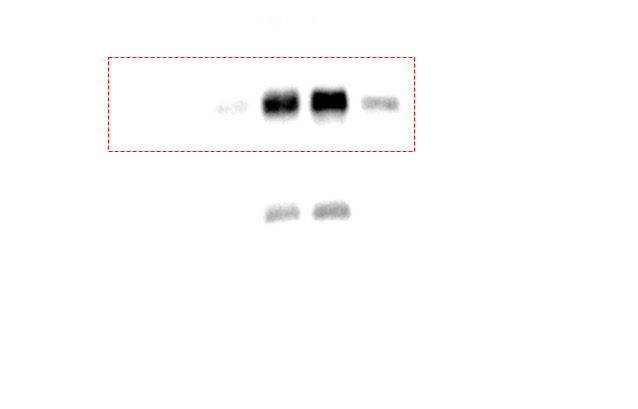

Supplement: Figure 1—figure supplement 2—source data 2. [file elife-83681-fig1-figsupp2-data2.zip › Figure 1 - figure supplement 2 - source data 2/upper left markup.jpg]

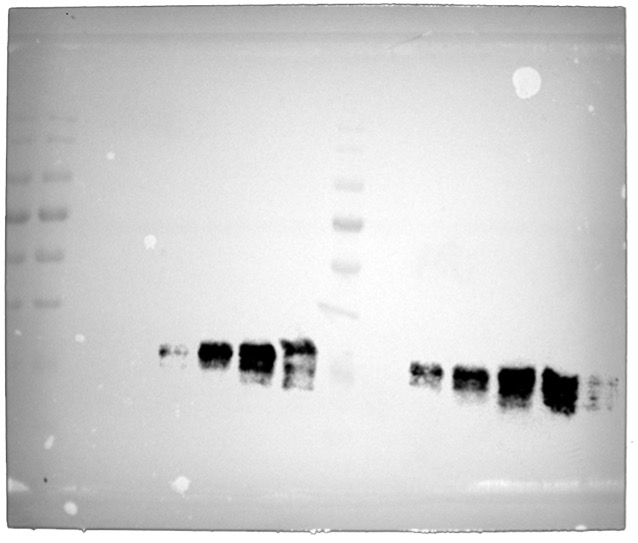

Supplement: Figure 1—figure supplement 2—source data 2. [file elife-83681-fig1-figsupp2-data2.zip › Figure 1 - figure supplement 2 - source data 2/upper and lower right.jpg]

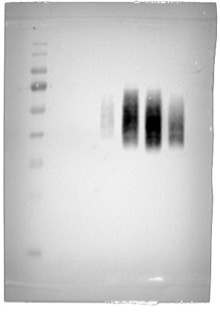

Supplement: Figure 1—figure supplement 2—source data 2. [file elife-83681-fig1-figsupp2-data2.zip › Figure 1 - figure supplement 2 - source data 2/upper middle.jpg]

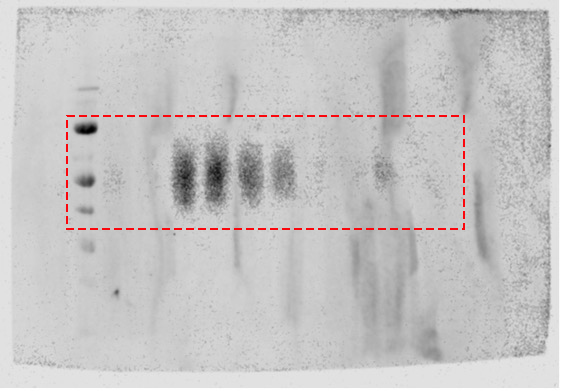

Supplement: Figure 2—source data 2. [file elife-83681-fig2-data2.zip › Figure 2 - source data 2/b inset markup.jpg]

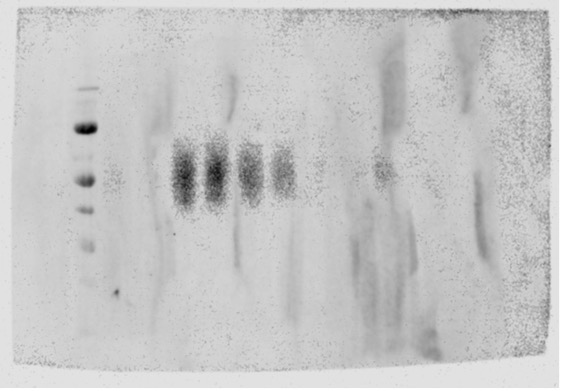

Supplement: Figure 2—source data 2. [file elife-83681-fig2-data2.zip › Figure 2 - source data 2/b inset.jpg]

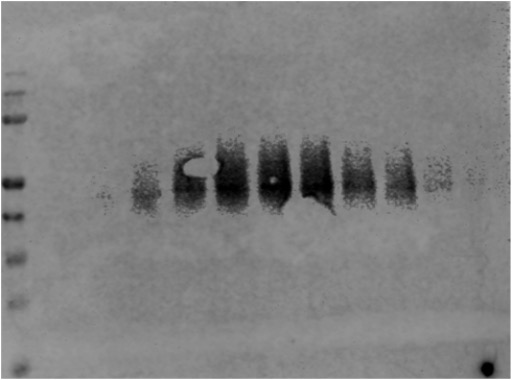

Supplement: Figure 2—figure supplement 1—source data 2. [file elife-83681-fig2-figsupp1-data2.zip › Figure 2 - figure supplement 1 - source data 2/a lower.jpg]

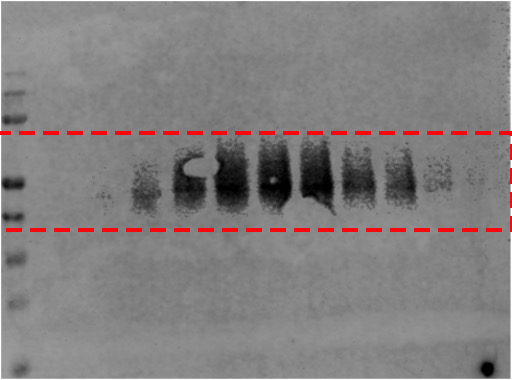

Supplement: Figure 2—figure supplement 1—source data 2. [file elife-83681-fig2-figsupp1-data2.zip › Figure 2 - figure supplement 1 - source data 2/a lower markup.jpg]

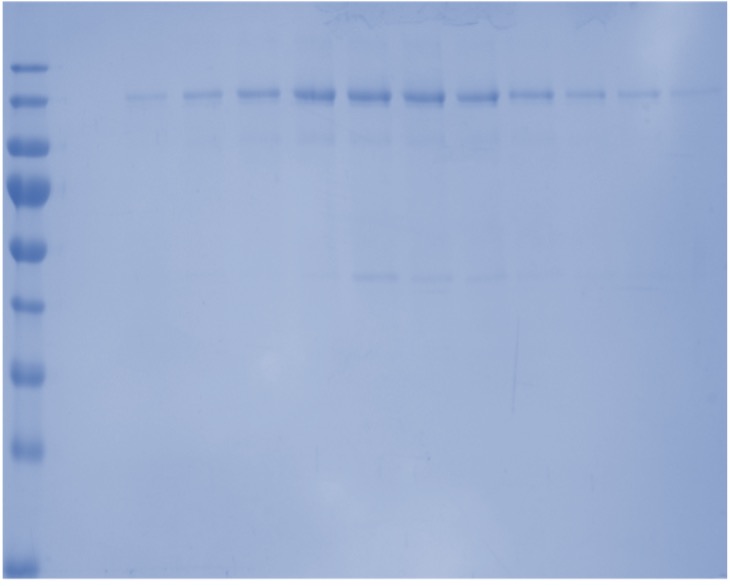

Supplement: Figure 2—figure supplement 1—source data 2. [file elife-83681-fig2-figsupp1-data2.zip › Figure 2 - figure supplement 1 - source data 2/a upper.jpg]

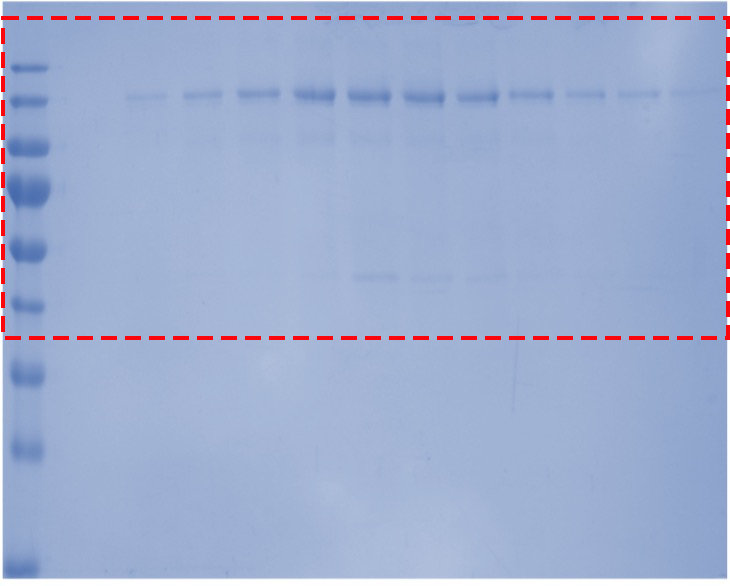

Supplement: Figure 2—figure supplement 1—source data 2. [file elife-83681-fig2-figsupp1-data2.zip › Figure 2 - figure supplement 1 - source data 2/a upper markup.jpg]

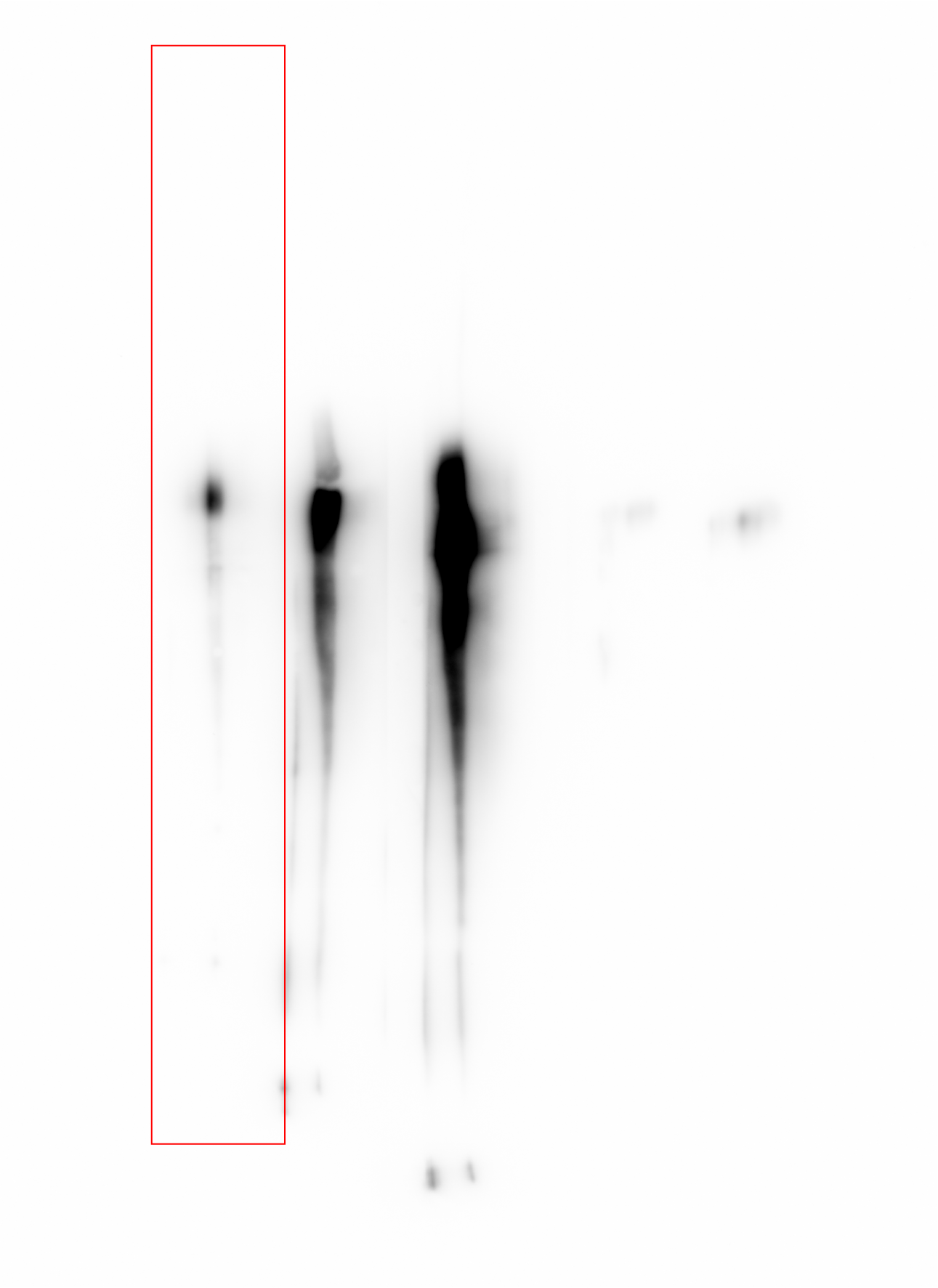

Supplement: Figure 2—figure supplement 2—source data 2. [file elife-83681-fig2-figsupp2-data2.zip › Figure 2 - figure supplement 2 - source data 2/a/PMCA upper markup.tif]

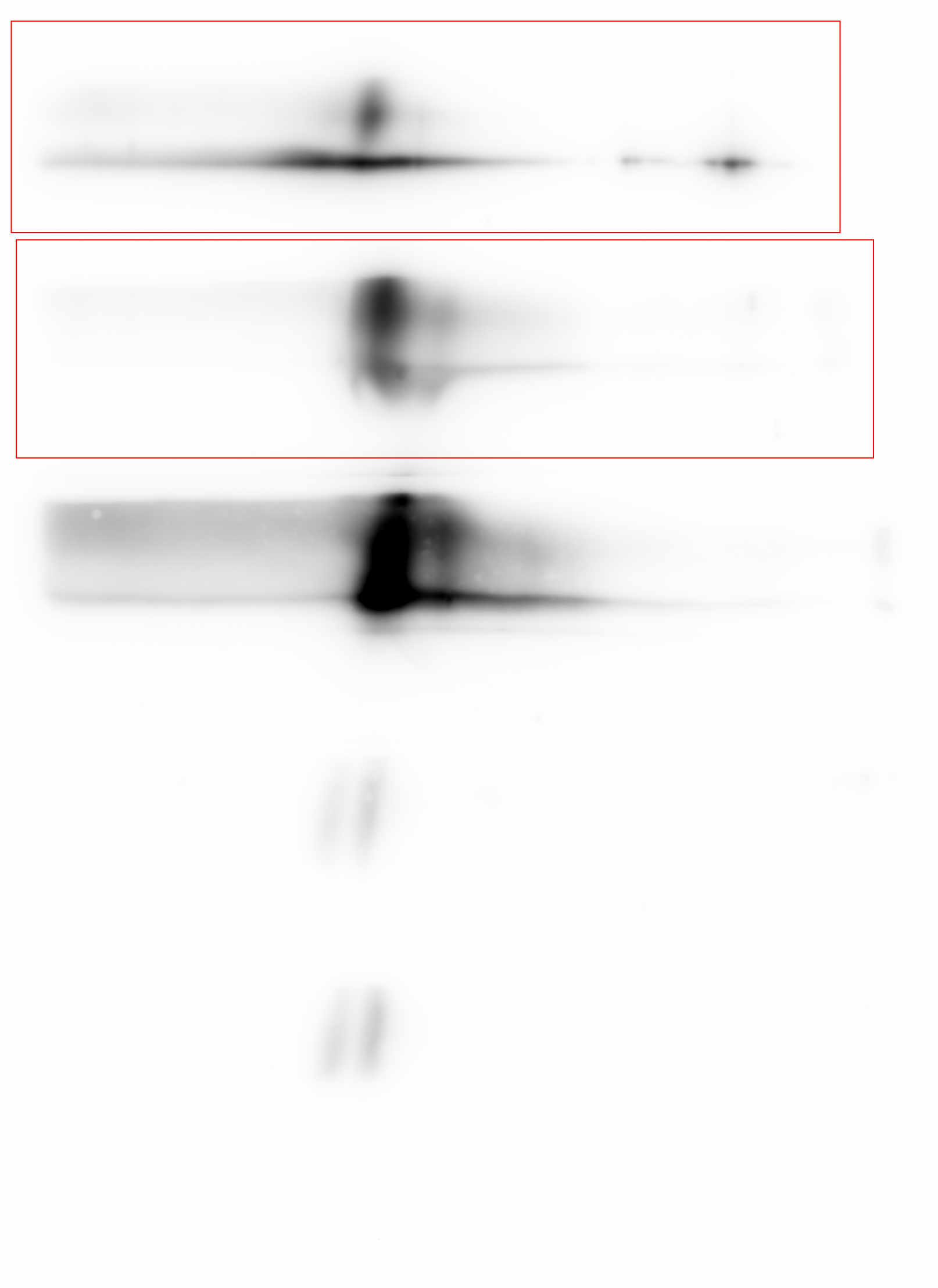

Supplement: Figure 2—figure supplement 2—source data 2. [file elife-83681-fig2-figsupp2-data2.zip › Figure 2 - figure supplement 2 - source data 2/a/basigin markup.tif]

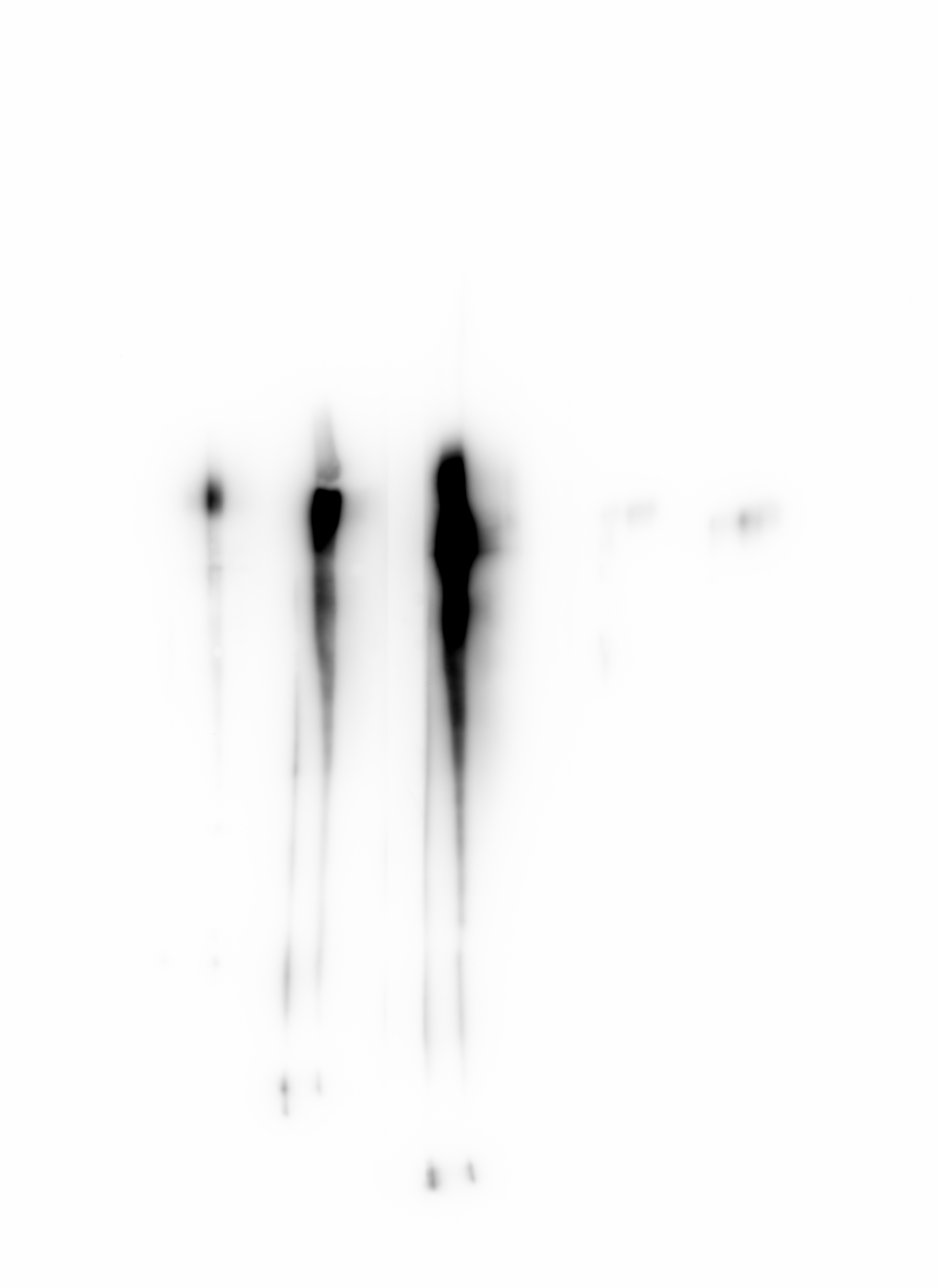

Supplement: Figure 2—figure supplement 2—source data 2. [file elife-83681-fig2-figsupp2-data2.zip › Figure 2 - figure supplement 2 - source data 2/a/PMCA upper.tif]

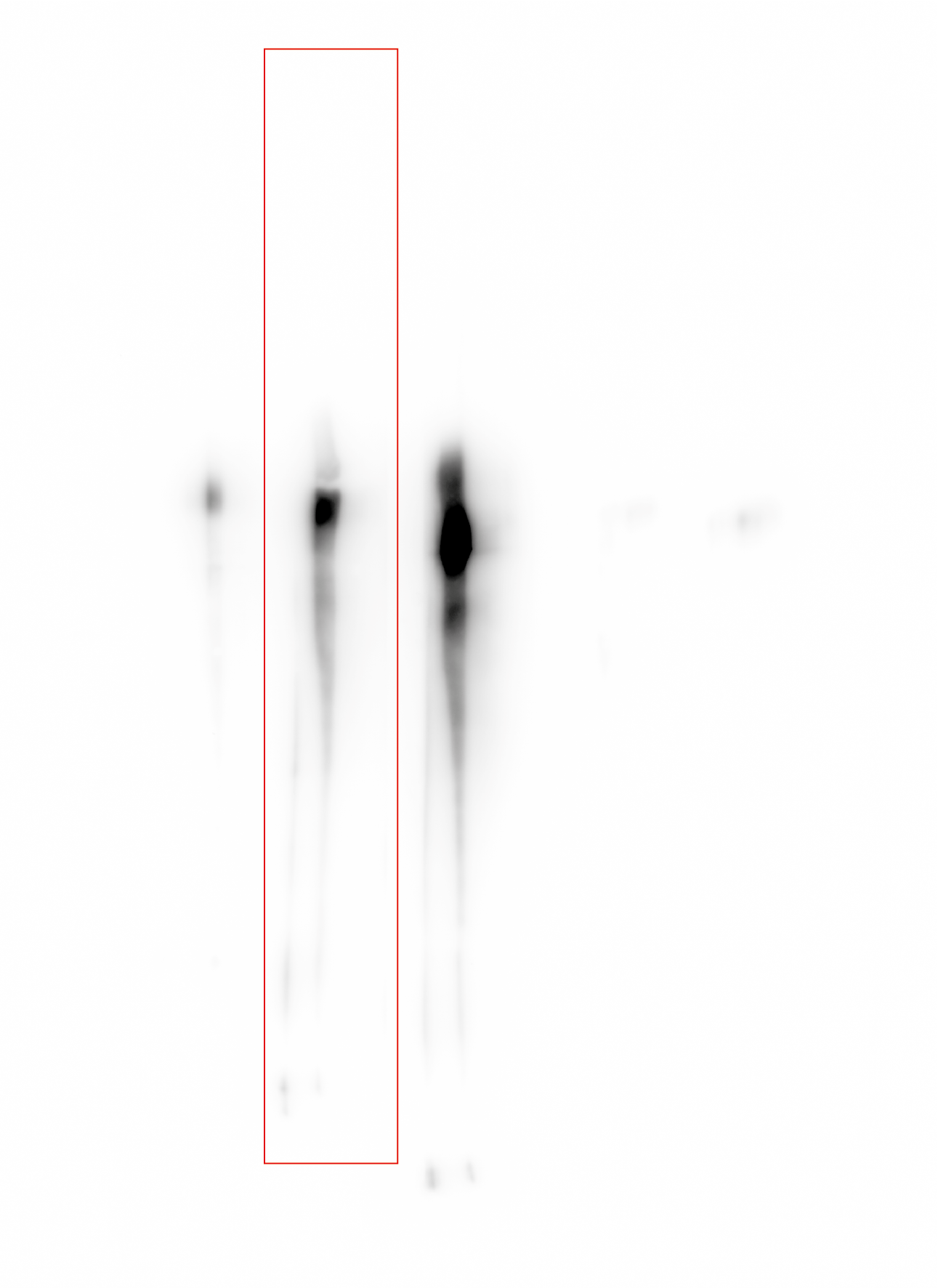

Supplement: Figure 2—figure supplement 2—source data 2. [file elife-83681-fig2-figsupp2-data2.zip › Figure 2 - figure supplement 2 - source data 2/a/PMCA lower markup.png]

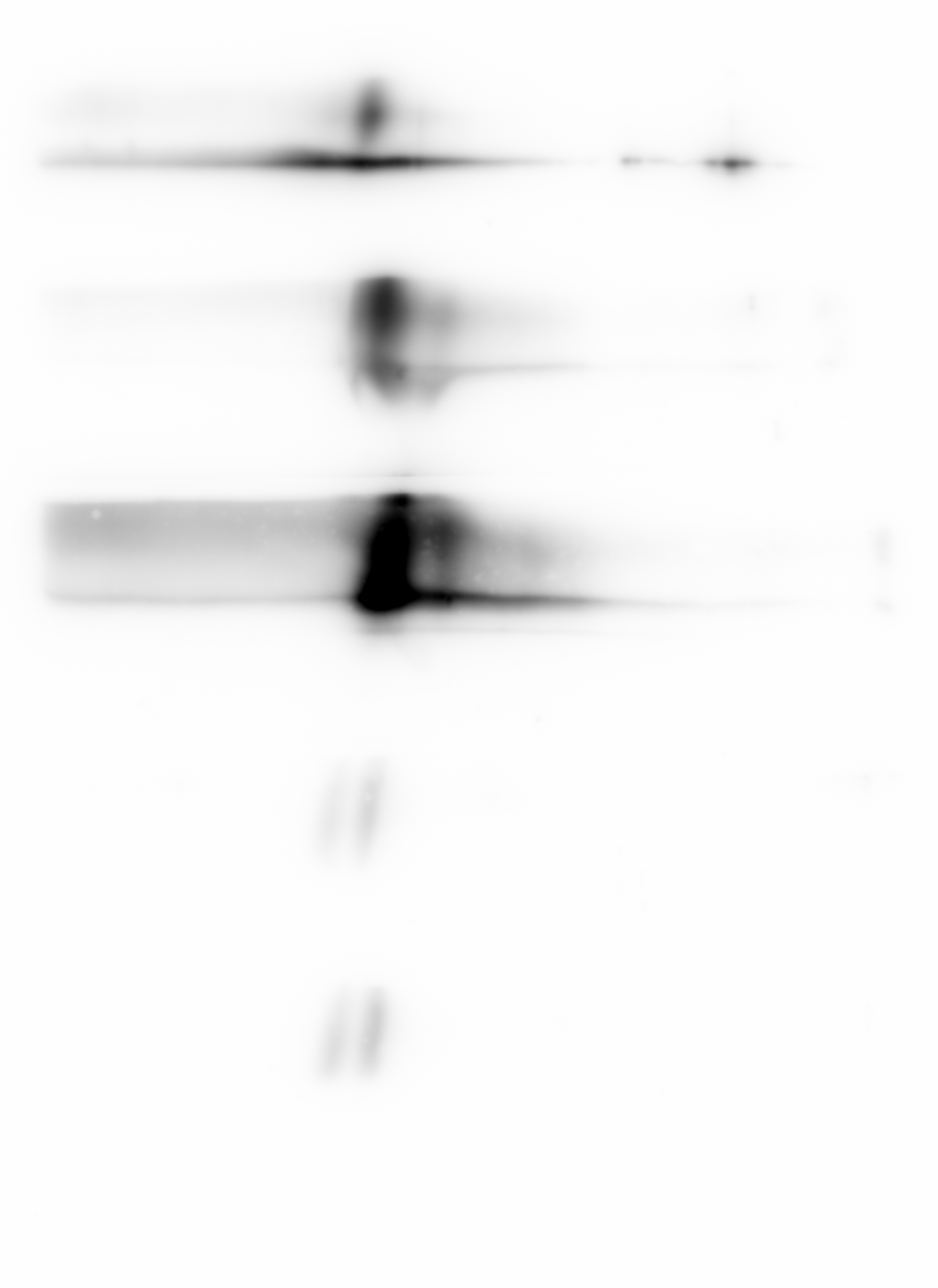

Supplement: Figure 2—figure supplement 2—source data 2. [file elife-83681-fig2-figsupp2-data2.zip › Figure 2 - figure supplement 2 - source data 2/a/Basigin.tif]

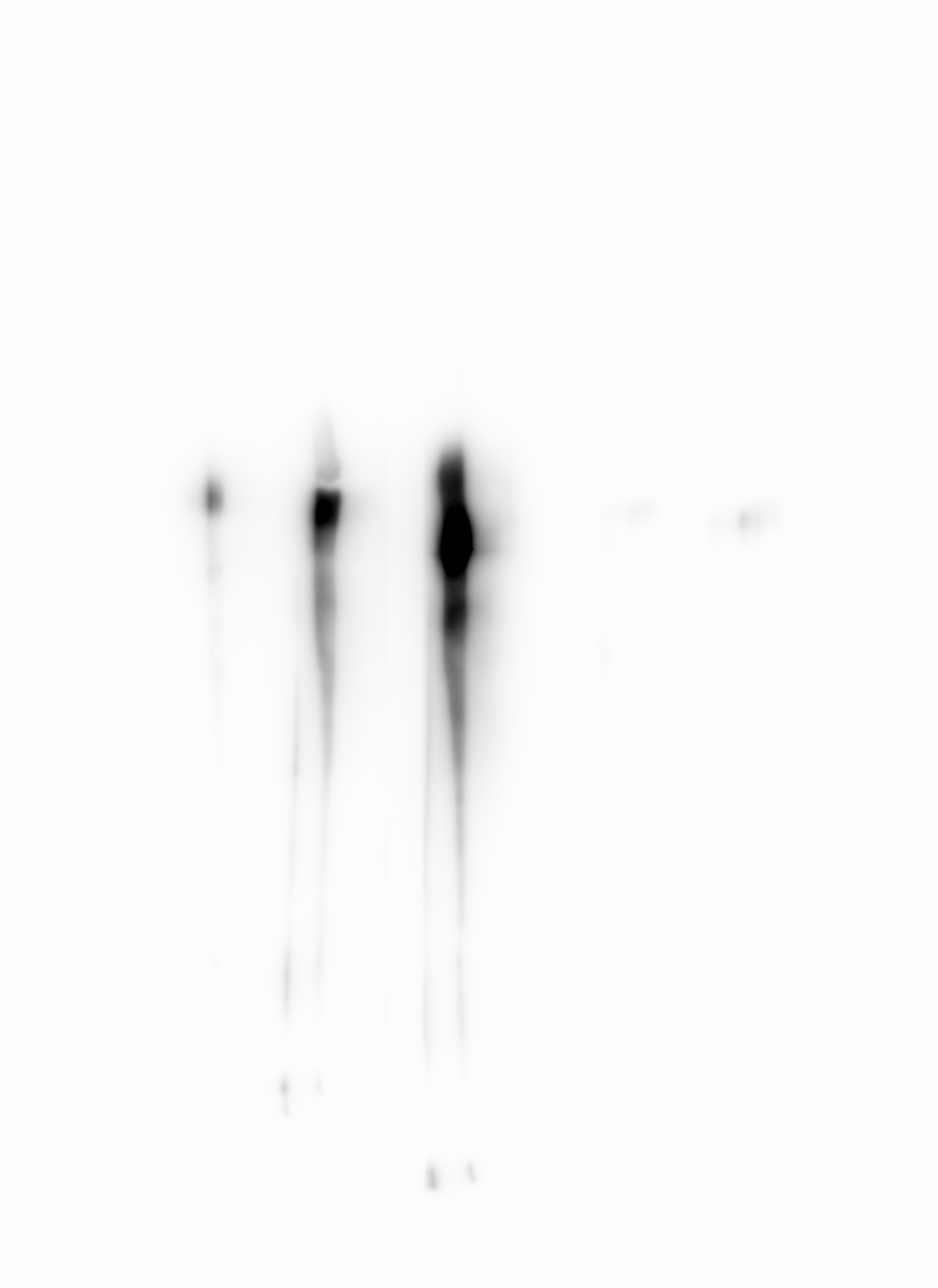

Supplement: Figure 2—figure supplement 2—source data 2. [file elife-83681-fig2-figsupp2-data2.zip › Figure 2 - figure supplement 2 - source data 2/a/PMCA lower markup.tif]

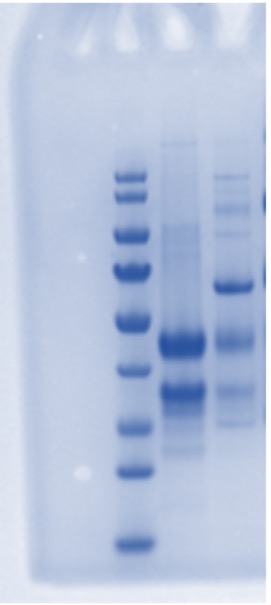

Supplement: Figure 3—source data 2. [file elife-83681-fig3-data2.zip › Figure 3 - source data 2/d inset.jpg]

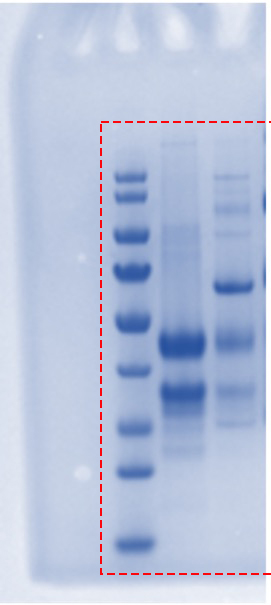

Supplement: Figure 3—source data 2. [file elife-83681-fig3-data2.zip › Figure 3 - source data 2/d inset markup.jpg]

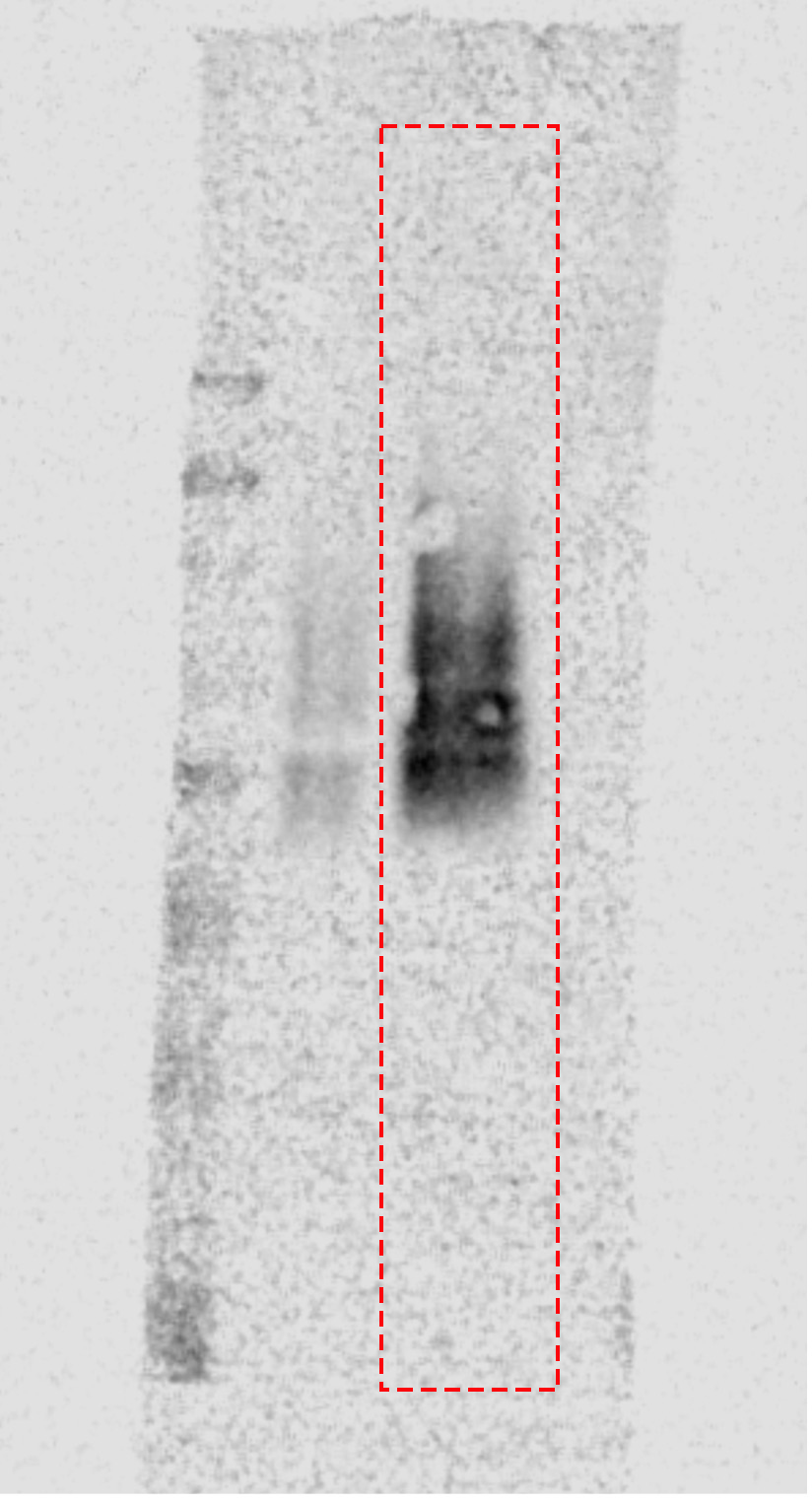

Supplement: Figure 3—figure supplement 2—source data 1. [file elife-83681-fig3-figsupp2-data1.zip › Figure 3 - figure supplement 2 - source data 2/bottom right markup.jpg]

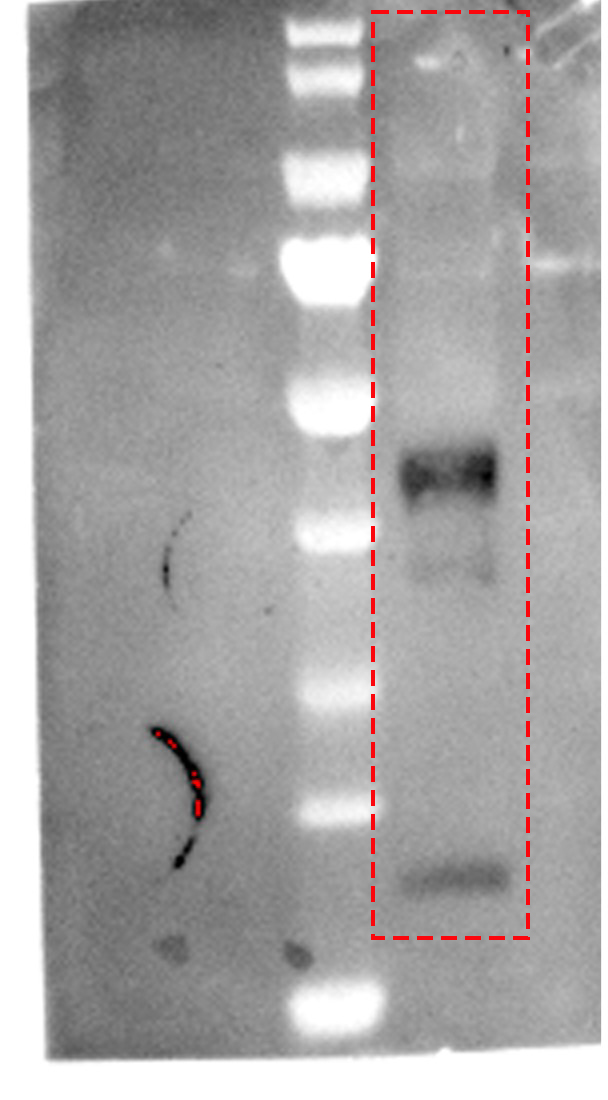

Supplement: Figure 3—figure supplement 2—source data 1. [file elife-83681-fig3-figsupp2-data1.zip › Figure 3 - figure supplement 2 - source data 2/top left markup.jpg]

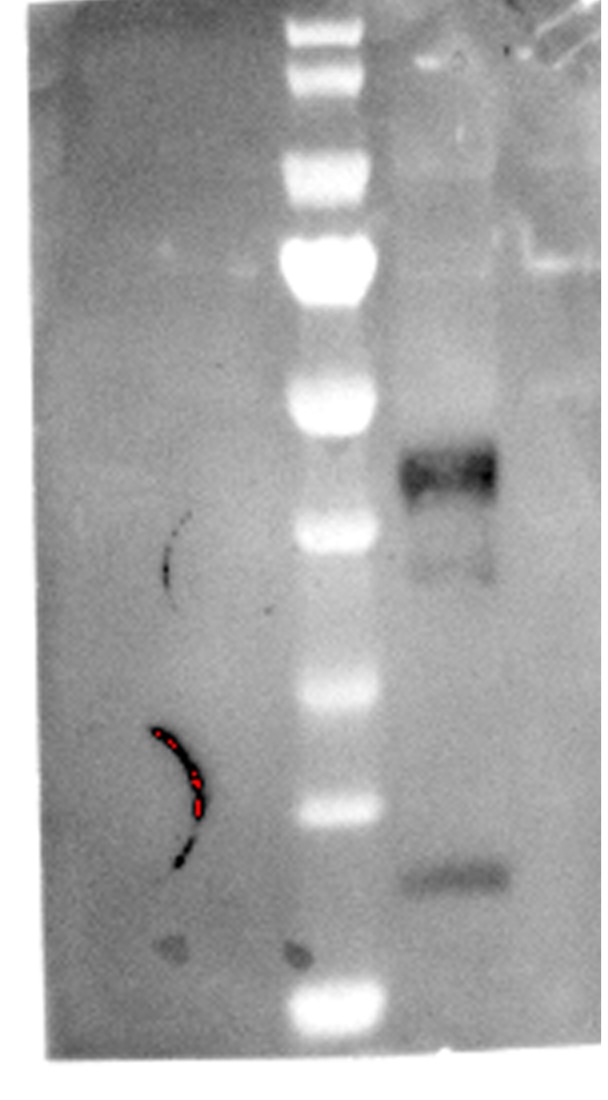

Supplement: Figure 3—figure supplement 2—source data 1. [file elife-83681-fig3-figsupp2-data1.zip › Figure 3 - figure supplement 2 - source data 2/top left.jpg]

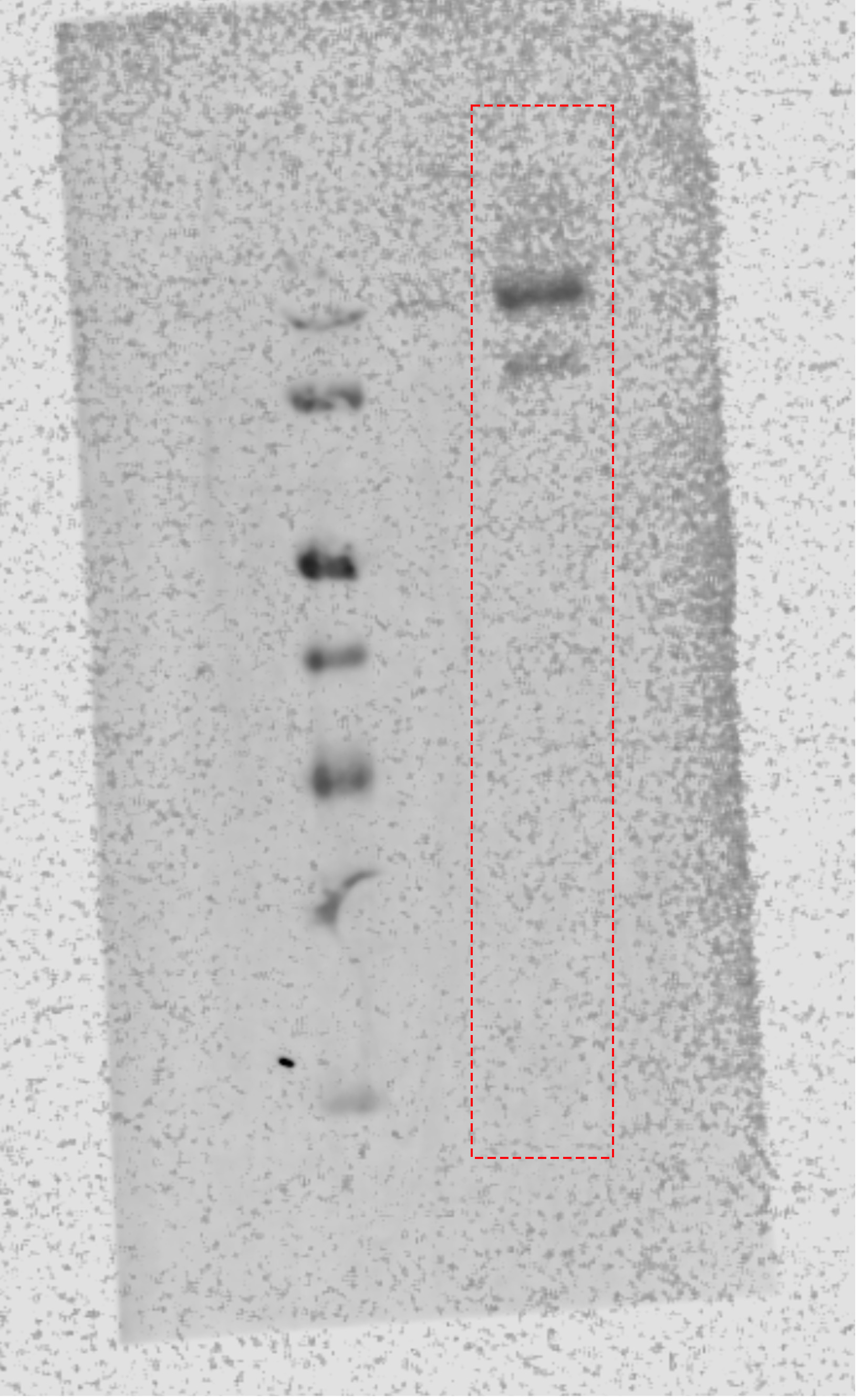

Supplement: Figure 3—figure supplement 2—source data 1. [file elife-83681-fig3-figsupp2-data1.zip › Figure 3 - figure supplement 2 - source data 2/top right markup.jpg]

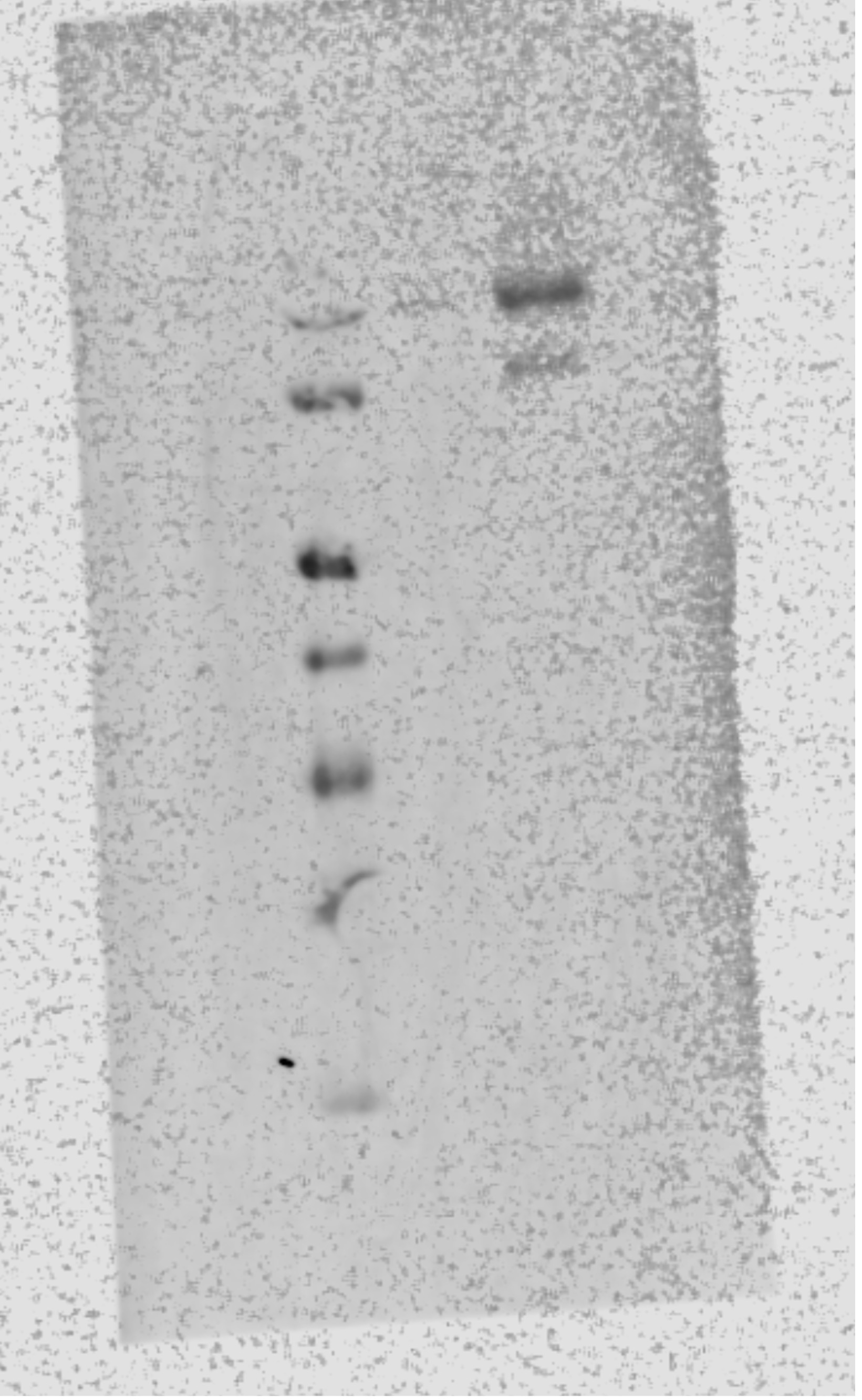

Supplement: Figure 3—figure supplement 2—source data 1. [file elife-83681-fig3-figsupp2-data1.zip › Figure 3 - figure supplement 2 - source data 2/top right.jpg]

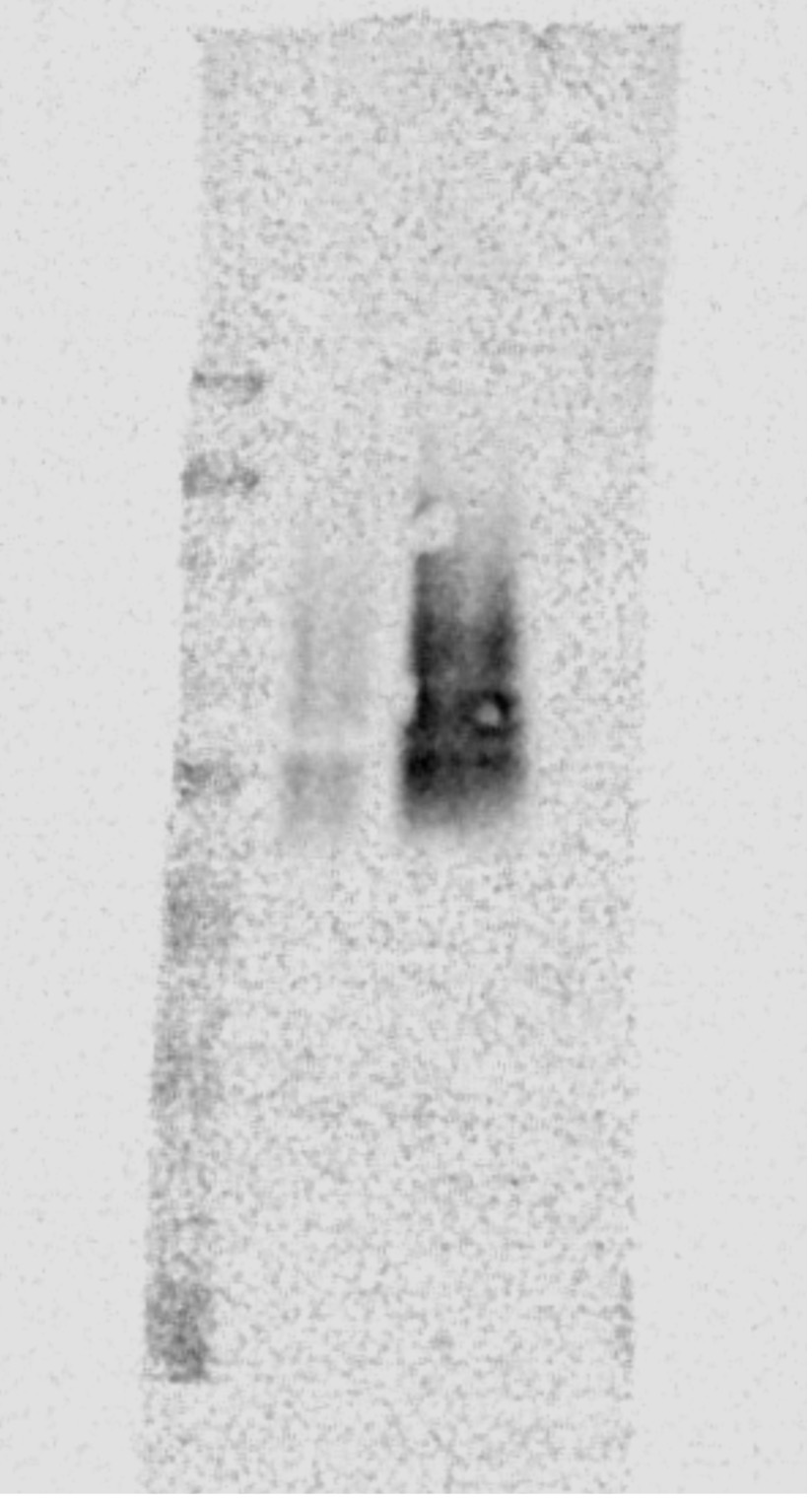

Supplement: Figure 3—figure supplement 2—source data 1. [file elife-83681-fig3-figsupp2-data1.zip › Figure 3 - figure supplement 2 - source data 2/bottom right.jpg]
