# Supplementary material for: Erythrocyte invasion-neutralising antibodies prevent Plasmodium falciparum RH5 from binding to basigin-containing membrane protein complexes
Source: eLife. 2023 Oct 5;12:e83681. doi: 10.7554/eLife.83681 (PMC10569788; doi:10.7554/eLife.83681)
Supplement: Supplementary file 1. [file elife-83681-supp1.docx]

**Supplementary File 1**. Binding constants derived from surface plasmon resonance analysis by fitting sensograms with Langmuir 1:1 model

| Analyte | k_a_ (M^-1^s^-1^) | k_d_ (s^-1^) | K_D_ (M) | R_max_ (RU) | χ^2^ (RU^2^) |
| --- | --- | --- | --- | --- | --- |
| Basigin ectodomain | 2.11 x 10^5^ | 0.166 | 7.86 x 10^-7^ | 79.98 | 2.44 |
| Basigin-PMCA | 9.55 x 10^4^ | 0.007188 | 7.938x10^-8^ | 85.01 | 7.71 |
| Basigin-MCT1 | 1.88 x 10^5^ | 0.008743 | 7.357 x 10^-8^ | 100.2 | 14.8 |
| Full length- Basigin | 1.54x10^5^ | 0.08721 | 5.65 x 10^-7^ | 70.52 | 0.71 |
