# Supplementary material for: Erythrocyte invasion-neutralising antibodies prevent Plasmodium falciparum RH5 from binding to basigin-containing membrane protein complexes
Source: eLife. 2023 Oct 5;12:e83681. doi: 10.7554/eLife.83681 (PMC10569788; doi:10.7554/eLife.83681)
Supplement: Supplementary file 2. [file elife-83681-supp2.docx]

**Supplementary File 2**. Binding constants derived from surface plasmon resonance analysis by fitting sensograms with two-state interaction model

| Analyte | k_a1_ (M^-1^s^-1^) | k_d1_ (s^-1^) | k_a2_ (M^-1^s^-1^) | k_d2_(s^-1^) | K_D_ (M) | R_max_ (RU) | χ^2^ (RU^2^) |
| --- | --- | --- | --- | --- | --- | --- | --- |
| Basigin-PMCA | 3.48x10^5^ | 0.0609 | 7.58x10^3^ | 6.74x10^-3^ | 8.78x10^-8^ | 98 | 1.85 |
| Basigin-MCT1 | 4.26x10^5^ | 0.0866 | 4.58x10^3^ | 3.88x10^-3^ | 9.31x10^-8^ | 131 | 3.09 |
